# From Berry to Bedside: Translational Potential of Berry-Derived Phytochemicals in HNSCC

**DOI:** 10.3390/molecules31111914

**Published:** 2026-06-02

**Authors:** Kishan Kumar Nyati, Ravi Ramalingam, Suvekshya Shrestha, Sushmitha Jagadeesha, Sonali Dasari, Shaheer Masood, Massar Yade, Parth A. Mehta, Shravya Kundety, Steve Oghumu

**Affiliations:** Department of Pathology, Pelotonia Institute for Immuno-Oncology, College of Medicine, The Ohio State University Wexner Medical Center, Columbus 43201, OH, USA; kishan.nyati@osumc.edu (K.K.N.); ravi.ramalingam@osumc.edu (R.R.); shrestha.119@buckeyemail.osu.edu (S.S.); jagadeesha.2@buckeyemail.osu.edu (S.J.); dasari.37@buckeyemail.osu.edu (S.D.); masood.45@buckeyemail.osu.edu (S.M.); yade.3@buckeyemail.osu.edu (M.Y.); mehta.696@buckeyemail.osu.edu (P.A.M.); kundety.2@buckeyemail.osu.edu (S.K.)

**Keywords:** head and neck squamous cell carcinoma, berries phytochemicals, natural compounds, tumor microenvironment, immunometabolism, chemoprevention, immune checkpoint inhibitors

## Abstract

Head and neck squamous cell carcinoma (HNSCC) remains an immunosuppressive and metabolically dysregulated malignancy, contributing to tumor progression and resistance to conventional therapies. Natural compounds offer a unique multi-target opportunity to address these challenges, with berry-derived phytochemicals emerging as particularly promising candidates. Preclinical evidence demonstrates that these compounds modulate dendritic cell activation, macrophage polarization, regulatory T cell function, and cytokine signaling, restoring immune balance while simultaneously regulating tumor metabolism and reducing chronic inflammation. Beyond these immunometabolic effects, berry-derived compounds influence glucocorticoid signaling at the endocrine–immune interface, alleviating additional immunosuppressive pressures within the tumor microenvironment. Early clinical studies support the feasibility of standardized berry-derived formulations as adjunctive agents. In patients with oral premalignant lesions and HNSCC, black-raspberry-based interventions including topical gels and oral troches, have demonstrated favorable safety profiles, measurable tissue uptake of bioactive phytochemicals, modulation of proliferation and inflammation-associated biomarkers (e.g., Ki-67, COX-2, and NF-κB), and partial histologic regression in a subset of lesions. Collectively, these pleiotropic actions highlight chemopreventive potential and provide a mechanistic rationale for combinatorial strategies with immune checkpoint inhibitors targeting PD-1/PD-L1 and CTLA-4. Opportunities for both local and systemic delivery may further enhance therapeutic efficacy. Integrating these natural compounds into precision chemoprevention and immunotherapy paradigms could inform rational drug discovery, biomarker-driven patient stratification, and combination therapy design. This review highlights the convergent immunologic, metabolic, and endocrine-targeted mechanisms of berry-derived phytochemicals in HNSCC and emphasizes their translational potential as integrative modulators of antitumor immunity.

## 1. Introduction

Head and neck squamous cell carcinoma (HNSCC) comprises epithelial malignancies of the oral cavity, pharynx, and larynx and remains a major global health burden. With nearly 380,000 new cases and >180,000 deaths annually, it is a leading cause of cancer mortality worldwide [1]. While tobacco, alcohol, and betel quid remain key drivers, human papillomavirus (HPV)-associated oropharyngeal cancers have reshaped epidemiology in Western populations [2,3]. Despite advances in surgery, radiotherapy, chemotherapy, targeted therapy, and immune checkpoint blockade, five-year survival remains approximately 50–60%, with significantly poorer outcomes in advanced disease [2,3,4]. These features highlight the need for mechanism-based therapeutic strategies capable of simultaneously targeting tumor signaling, inflammation, and immune dysfunction.

These limited gains reflect the biological complexity of HNSCC. Tumor progression is driven not only by genetic alterations but also by a dynamic tumor microenvironment (TME) characterized by chronic inflammation, immune suppression, and metabolic reprogramming. Persistent NF-κB and STAT3 activation, together with dysregulated cytokine and growth factor signaling, promotes tumor survival and therapy resistance [5]. In parallel, immune dysfunction marked by impaired dendritic cells (DC), expanded regulatory T (Treg) cells, accumulation of myeloid-derived suppressor cells (MDSCs), and reduced cytotoxic T cell activity further sustain disease progression [5]. Although PD-1/PD-L1 blockade has transformed treatment, durable responses are limited to a subset of patients, highlighting the need for strategies that reprogram the TME rather than target single pathways. 

Natural products remain central to anticancer drug discovery, contributing to more than half of approved agents or their scaffolds [6]. Their key advantage lies in polypharmacology, enabling simultaneous modulation of multiple signaling pathways particularly relevant in HNSCC, where redundant signaling networks drive therapeutic resistance. Among these, berries are rich sources of bioactive phytochemicals, including anthocyanins, ellagitannins, flavonols, and phenolic acids such as protocatechuic acid (PCA) [7,8,9]. These phytochemical classes are structurally defined by distinct polyphenolic scaffolds, including flavonoid C6-C3-C6 backbones (flavonols and anthocyanidins), hydrolysable galloyl/hexahydroxydiphenoyl (HHDP) units (ellagitannins), and stilbene-based frameworks, with functional activity strongly influenced by hydroxylation and glycosylation patterns that determine redox potential, receptor binding affinity, and cellular uptake. Berries also contain less explored nitrogen containing bioactive molecules such as polyamines and spermine derived conjugates that may contribute to immune regulation, redox homeostasis, and anticancer activity [10,11]. Importantly, berry-derived phytochemicals represent chemically diverse molecular entities that can be categorized into distinct structural subclasses with known structure–activity relationships (SAR), where variations in substitution patterns, glycosylation, and polymerization degree influence their ability to interact with kinases, transcription factors, and membrane receptors involved in oncogenic signaling [7,8,9,12]. Berry-derived molecules, their rich extracts or their derived metabolites also exhibit pleiotropic biological activities that extend beyond antioxidant effects to include regulation of transcription factors, kinases, inflammatory mediators, and metabolic pathways. Such properties position berry-derived phytochemicals as candidate modulators of the complex signaling networks that drive HNSCC pathogenesis.

Beyond antioxidant activity, these compounds regulate transcriptional programs, kinases, inflammatory mediators, and metabolic pathways, positioning them as multi-target modulators of HNSCC biology. Preclinical studies support these effects across cancer models, including colorectal and breast cancer, where berry-derived compounds suppress tumor signaling, inhibit proliferation, and induce apoptosis [13,14]. In HNSCC, the oral cavity provides a unique site for direct exposure, making berries particularly relevant for chemopreventive investigation in preclinical and early translational studies. Black raspberries (BRB; *Rubus occidentalis*) have shown consistent suppression of inflammation-driven carcinogenesis and epithelial transformation in preclinical oral cancer models [15]. Mechanistically, these effects are associated with modulation of COX-2, NF-κB, and EGFR signaling, along with regulation of oxidative DNA damage and epithelial-mesenchymal transition (EMT)-related gene networks [15,16]. Beyond epithelial effects, berry phytochemicals modulate immune responses, including DC cell activation, antigen presentation, and T cell function [17,18]. They also influence the balance between effector and Tregs, a key determinant of immune suppression in HNSCC [19]. This positions them as potential modulators of immunotherapy responsiveness in preclinical and early translational contexts. In parallel, these compounds regulate metabolic pathways central to tumor–immune interactions, including glycolysis and adenosine monophosphate-activated protein kinase (AMPK) signaling, linking dietary phytochemicals to cancer immunometabolism [20]. Additional evidence suggests effects on glucocorticoid (GC) signaling, further expanding their impact on inflammatory and immune regulation [21].

Collectively, these findings challenge the view of dietary phytochemicals as passive antioxidants, instead supporting a model in which berry-derived compounds act as active immunometabolic regulators. By targeting interconnected inflammatory, immune, and metabolic networks, they align with network pharmacology principles and multi-target therapeutic strategies. From a chemical biology perspective, this multi-target activity is enabled by structural diversity across berry phytochemicals, allowing differential binding to proteins involved in kinase signaling, transcriptional regulation, and redox-sensitive pathways. Despite promising data, clinical translation is limited by low bioavailability, rapid metabolism, compositional variability, and lack of standardized formulations. Advances in delivery systems, metabolite identification, and clinical validation are needed to bridge dietary exposure and therapeutic application. 

In this review, we present evidence on berry-derived phytochemicals in HNSCC, focusing on their roles in immune and metabolic regulation, chemoprevention, and therapeutic development. We propose a unified framework in which these compounds are viewed as metabolically active multi-component systems that collectively shape the TME. We organize this synthesis into three axes: (i) immune modulation, (ii) metabolic regulation, and (iii) GC and inflammatory signaling, providing a structured basis for interpreting current evidence and identifying translational opportunities. 

## 2. Natural Products as Drug Discovery Platforms

Building on the recognition that HNSCC is sustained by dynamic interactions among inflammatory, immune, metabolic, and stromal networks, there is increasing interest in therapeutic strategies that extend beyond single-pathway inhibition. HNSCC is characterized by substantial molecular heterogeneity, including EGFR amplification, PI3K/AKT pathway activation, TP53 loss-of-function mutations, hypoxia-driven signaling, and immune suppression within the TME. These interconnected alterations contribute to therapeutic resistance and limit the long-term efficacy of pathway-selective interventions. Consequently, therapeutic approaches capable of simultaneously modulating multiple oncogenic and immunometabolic pathways may provide greater durability than conventional single-target strategies.

Within this framework, natural products represent a valuable and underexplored resource for oncology drug discovery. Unlike highly selective synthetic inhibitors, many naturally occurring compounds exhibit coordinated multi-target activity, enabling simultaneous regulation of inflammatory signaling, oxidative stress responses, metabolic adaptation, angiogenesis, and immune function. This systems-level pharmacology is particularly relevant in HNSCC, where compensatory pathway activation frequently undermines targeted therapies. Berry-derived phytochemicals, including anthocyanins, ellagitannins, flavonols, and phenolic acids, have emerged as promising modulators of signaling pathways implicated in HNSCC progression, including EGFR/PI3K/AKT, NF-κB, STAT3, HIF-1α, VEGF, and PD-L1-associated immune suppression. The following sections examine how berry-derived natural products can be integrated into modern drug discovery paradigms, with emphasis on mechanistic signaling, network modulation, and translational relevance in HNSCC.

### 2.1. From Single-Target Inhibition to Network Modulation

Despite advances in targeted therapy, single-target approaches remain limited in biologically heterogeneous malignancies such as HNSCC. Extensive signaling redundancy, pathway crosstalk, compensatory feedback activation, and metabolic plasticity enable tumor cells to evade selective therapeutic pressure and develop resistance. EGFR-targeted therapies, for example, frequently demonstrate limited durability due to activation of alternative proliferative pathways including PI3K/AKT, STAT3, MAPK, and NF-κB signaling. Furthermore, interactions between malignant cells, stromal populations, and immune cells within the TME dynamically influence therapeutic response and contribute to immune evasion. Immune checkpoint blockade further illustrates the complexity of HNSCC biology. Although PD-1 inhibitors improve survival in a subset of patients, response rates remain modest, emphasizing that tumor progression is sustained by multiple interconnected inflammatory, metabolic, and immunological pathways rather than a single dominant axis [6]. Consequently, there is increasing interest in therapeutic strategies capable of simultaneously modulating multiple components of tumor-promoting networks.

Natural products are particularly well suited to this paradigm. As evolutionary products of chemical defense, plant-derived compounds frequently interact with diverse molecular targets and signaling systems. Berry-derived phytochemicals provide a representative example of such network-oriented pharmacology. Anthocyanins, ellagitannins, flavonols, and phenolic acids collectively regulate inflammatory signaling, oxidative stress, metabolic adaptation, angiogenesis, and immune cell activation. Mechanistically, these compounds suppress NF-κB and STAT3 inflammatory signaling, inhibit EGFR/PI3K/AKT activation, reduce VEGF-mediated angiogenesis, modulate AMPK-associated metabolic pathways, and attenuate ROS-dependent signaling cascades [15,22]. Importantly, several berry-derived compounds demonstrate mechanistic activity directly relevant to HNSCC progression. Cranberry-derived proanthocyanidins suppress MMP-2/MMP-9 activity and restore E-cadherin expression, thereby inhibiting EMT and invasive phenotypes in oral squamous carcinoma models. Delphinidin inhibits EGFR- and VEGFR2-associated signaling while reducing PI3K/AKT and MAPK phosphorylation. BRB-derived anthocyanins suppress COX-2, IL-6, VEGF, and NF-κB activation in 4-nitroquinoline 1-oxide (4NQO)- and 7,12-dimethylbenzanthracene (DMBA)-driven oral carcinogenesis models, simultaneously reducing dysplasia progression, angiogenesis, and inflammatory cytokine production [15,17]. Collectively, these observations support a shift toward network-based therapeutic strategies that more effectively address the biological complexity of HNSCC. By modulating multiple interconnected pathways, natural products and berry-derived compounds in particular offer a complementary investigational approach to conventionally targeted therapies and a foundation for the development of more integrative anticancer interventions.

### 2.2. Toward a Systems-Level Framework for Phytochemical Drug Discovery

Cancer is increasingly recognized as a systems-level disease, and HNSCC exemplifies this complexity: single-cell transcriptomic analyses of HNSCC tumors reveal co-existing malignant, immune, and stromal cell states with distinct signaling dependencies, making global network modulation more effective than targeting a single node [5,23]. HPV-positive and HPV-negative HNSCC further display fundamentally different immune landscapes, inflammatory signaling patterns, and metabolic phenotypes, complicating therapeutic targeting strategies focused on individual molecular nodes. Within this framework, berry-derived phytochemical mixtures historically dismissed as pharmacologically imprecise are now being characterized as structured, multi-component modulators of these biological networks.

Berries provide a representative example of this paradigm. They contain diverse and interacting phytochemicals whose collective activity generates biological effects that are not readily predictable from individual constituents. As mentioned, major classes, including anthocyanins, ellagitannins, and phenolic acids, exhibit complementary and partially overlapping functions, enabling coordinated regulation of immune and metabolic processes. For example, BRB-derived compounds and their metabolite PCA modulate DC activation and influence downstream T cell responses, while whole berry extracts alter antigen specific immune activity [17,18,19]. These findings highlight integrated regulation of innate and adaptive immunity rather than discrete pathway inhibition.

Metabolomic and functional studies further support this systems-level perspective. BRB supplementation has been shown to alter glycolytic intermediates and engage AMPK-dependent metabolic signaling during oral carcinogenesis, linking metabolic reprogramming with immune modulation [20]. In parallel, modulation of local GC signaling suggests additional layers of crosstalk between endocrine and immune pathways within the TME [21].

Recent advances in transcriptomics, metabolomics, and computational modeling now enable more comprehensive mapping of these complex interactions. Rather than isolating single active compounds, these approaches facilitate identification of synergistic phytochemical networks and their coordinated effects across interconnected pathways. Such insights support a conceptual framework in which natural products function as intrinsic combinatorial systems, offering a foundation for the rational design of multi-target investigational strategies in HNSCC.

### 2.3. Advantages of Natural Products in Chemoprevention

Effective chemoprevention requires agents that are safe for long-term use, capable of intercepting early carcinogenic events, and broadly effective across heterogeneous populations. Natural products, particularly dietary phytochemicals, possess characteristics that may support their investigation as chemopreventive agents. First, their long history of dietary exposure provides an established safety profile. For example, BRB have demonstrated favorable tolerability in preclinical and limited early-phase investigational studies [18]. Second, their pleiotropic activity enables simultaneous modulation of inflammatory, immune, and metabolic pathways that are disrupted early in carcinogenesis. Chronic inflammation is a key driver of oral epithelial transformation, and berry-derived compounds attenuate pro-inflammatory mediators while restoring immune homeostasis [17,18,19]. Third, distributed network modulation may reduce the possibility of resistance. Unlike single-target therapies, which impose strong selective pressure on discrete molecular nodes, multi-pathway modulation limits adaptive escape. For example, preclinical studies of berry-derived interventions demonstrate concurrent modulation of Treg activity and metabolic reprogramming, supporting this principle [9,13]. Finally, the oral cavity offers a unique pharmacologic advantage for HNSCC prevention. Direct mucosal exposure during ingestion permits higher local concentrations of bioactive compounds, potentially enhancing efficacy while minimizing systemic toxicity. Collectively, these features support continued investigation of natural products as potential long-term chemopreventive strategies in HNSCC.

### 2.4. Berries as Multi-Component Pharmacological Libraries

Having their advantages in chemoprevention, berries can also be conceptualized as complex, multi-component pharmacological libraries that provide a rich resource for drug discovery. Rather than being mere dietary supplements, berries comprise structurally diverse bioactive molecules with overlapping and complementary molecular targets. Each species contains hundreds of phytochemicals. Of those, anthocyanins-rich preparations and their metabolically-derived phenolic products modulate redox-sensitive transcription factors, ellagitannins regulate inflammatory cascades, and phenolic acids such as PCA influence immune cell activation [18]. Whole berry extracts further impact antigen presentation and T cell priming, reflecting coordinated effects on both innate and adaptive immunity [19].

From a drug discovery perspective, this chemical diversity represents a strategic advantage. Natural matrices provide pre-assembled combinations of compounds that may exhibit synergistic activity, enhanced stability, and broader network coverage than single synthetic agents. Modern analytical and high throughput platforms now enable systematic characterization of these mixtures, facilitating identification of bioactive fractions and lead candidates. Preclinical evidence from oral carcinogenesis models demonstrates coordinated modulation of immune responses, metabolic pathways, inflammatory signaling, and endocrine regulation [17,18,19,20,21]. Such multi-dimensional activity is rarely achievable with single-target synthetic drugs without increasing toxicity, underscoring the investigational potential of berry-derived natural products as multi-functional pharmacological platforms.

### 2.5. Challenges in Translational Development

While berry-derived phytochemicals offer compelling multi-target activity and chemopreventive potential, several challenges must be addressed to enable their translation into clinical applications. Bioavailability and pharmacokinetics present a major limitation. Many polyphenols are rapidly metabolized and eliminated, and their systemic activity often depends on microbiota-derived metabolites, introducing significant interindividual variability. Strategies such as nanoformulation, encapsulation, or structural modification are being explored to enhance stability, tissue distribution, and pharmacological efficacy. Recent advances in nanotechnology-based delivery systems have significantly improved the pharmacological feasibility of dietary polyphenols. Liposomal encapsulation, polymeric nanoparticles, and lipid-based carriers have been shown to enhance stability, tissue targeting, and systemic exposure of flavonoids including anthocyanins and resveratrol, thereby addressing long-standing limitations in translational application [12,16]. Standardization and quality control are equally critical. Phytochemical composition varies with species, environmental conditions, and processing methods. Rigorous standardization, coupled with quantitative profiling using metabolomics and high-resolution mass spectrometry, is essential to ensure reproducibility, regulatory compliance, and identification of active fractions. Regulatory pathways pose additional hurdles. Translating natural products from dietary supplements to therapeutics requires adherence to stringent frameworks, including demonstration of safety, efficacy, and manufacturing consistency under good manufacturing practice (GMP) standards. Finally, mechanistic deconvolution remains a scientific challenge. While multi-target activity underpins the therapeutic advantage of berry phytochemicals, it also complicates the identification of dominant mechanisms of action. Integrating systems biology approaches with targeted experimental validation is essential to define key drivers of efficacy without oversimplifying the complex network of interactions. Collectively, addressing these challenges is critical to bridge the gap between preclinical promise and clinical implementation, ensuring that the full potential of berry-derived natural products can be realized in HNSCC prevention and therapy.

### 2.6. Integrating Natural Product Libraries into Modern Oncology

The multifaceted biological activity of berry-derived phytochemicals, combined with advances in systems biology and high throughput screening, positions natural product libraries as a valuable complement to contemporary oncology drug discovery. Natural products have repeatedly transitioned from traditional use to clinically transformative therapeutics [6], and berries exemplify this potential as structured, multi-component pharmacological platforms. Integration of these compounds into modern therapeutic pipelines requires approaches that leverage their network targeting properties while addressing challenges in standardization, bioavailability, and mechanistic clarity.

Preclinical evidence suggests that natural product libraries can be systematically characterized to identify synergistic combinations that simultaneously modulate inflammatory, immune, and metabolic pathways. Berry-derived compounds enhance immune surveillance by modulating DC activation and downstream T cell responses [17,18] and influence Tregs balance within the TME [19]. Concurrently, these compounds affect metabolic reprogramming, including glycolytic intermediates and AMPK-associated pathways [15,20], providing a mechanistic link between immune modulation and metabolic adaptation. Computational modeling, network pharmacology, and chemical analytics [5] enable systematic mapping of these interactions, guiding identification of bioactive fractions and rational combinations suitable for translational development. Beyond chemoprevention, berry-derived compounds are being investigated for their potential to complement existing therapies. Their reported ability in preclinical models to modulate immune responses, suppress pro-inflammatory signaling, and regulate endocrine pathways [19,21] suggests potential synergy with immunotherapy, targeted therapy, and conventional cytotoxic agents. Incorporating natural product libraries into investigational combination regimens could potentially expand therapeutic windows, improve efficacy, and mitigate resistance, particularly in biologically heterogeneous malignancies such as HNSCC. Finally, translating natural product libraries into oncology requires a framework that unites preclinical systems-level evidence, mechanistic understanding, and rigorous clinical evaluation. Standardized formulations, validated biomarkers of activity, and adaptive trial designs are essential to realize their full therapeutic potential. By bridging dietary chemoprevention and precision pharmacology, berry-derived natural product libraries exemplify a model for next generation, network-oriented therapeutics capable of addressing the complexity of modern cancer biology.

## 3. Phytochemical Classes and Structure–Activity Relationships of Berry-Derived Compounds Relevant to HNSCC

Berries are distinguished from other dietary sources and medicinal plants by a characteristic combination of phytochemical classes and their coordinated metabolic fate, rather than the presence of a single dominant bioactive compound. Compared to most fruits and botanicals, berries contain exceptionally high levels and structural diversity of anthocyanins, alongside significant quantities of ellagitannins, flavonols, and phenolic acids. These phytochemical classes are defined by distinct molecular scaffolds, including flavonoid C6-C3-C6 backbones (anthocyanins and flavonols), hydrolysable galloyl/ HHDP ester structures (ellagitannins), and simple phenylpropanoid-derived phenolic acids, where structural features such as hydroxylation pattern, glycosylation, and degree of polymerization critically determine receptor interactions, redox activity, and signaling modulation in cancer cells. Importantly, these compounds function within a metabolically integrated system, in which parent phytochemicals undergo rapid biotransformation following ingestion. For example, anthocyanins are extensively metabolized into low molecular weight phenolic acids such as PCA, while ellagitannins are converted by the gut microbiota into urolithins, which exhibit greater systemic persistence and bioactivity [24,25]. This metabolic conversion is now recognized as a key determinant of SAR, where bioactivity is often mediated by metabolite scaffolds rather than parent molecules, particularly for high molecular weight polyphenols with limited bioavailability.

This predictable and efficient conversion into bioavailable metabolites distinguishes berry polyphenols from many phytochemicals found in medicinal herbs, which often exhibit more limited or variable metabolic activation. In addition, berries provide these compounds within a complex and reproducible phytochemical matrix, enabling synergistic interactions that simultaneously target inflammatory signaling, redox balance, and immune regulation. Such multi-component activity contrasts with the single-target paradigm often associated with isolated natural products. This synergy arises from complementary structural diversity across phytochemical subclasses, enabling simultaneous modulation of kinases, transcription factors, and membrane-associated receptors involved in HNSCC signaling networks. Collectively, these features position berries as functionally distinct dietary sources, whose biological effects arise from the interplay between their unique composition and metabolite-driven mechanisms of action. Understanding the composition and tissue bioavailability of these compounds is critical for designing mechanism-driven chemopreventive and investigational therapeutic strategies. The major berry-derived bioactive compounds, their molecular targets, and validated effects in HNSCC are summarized in Table 1.

### 3.1. Anthocyanins

Anthocyanins are a prominent subclass of flavonoids widely distributed in berries such as blueberries, blackberries, strawberries, and grapes, and are largely responsible for their characteristic pigmentation. These compounds have attracted considerable attention due to their diverse biological activities and potential therapeutic relevance [26]. Structurally, anthocyanins differ in the number and position of hydroxyl and methoxy groups on the B ring, as well as in their glycosylation patterns, features that critically influence their stability, redox properties, and bioactivity [27]. In general, increased hydroxylation enhances radical scavenging capacity, linking chemical structure to antioxidant potential. Importantly, these structural modifications also influence binding affinity toward signaling proteins such as EGFR, VEGFR2, and NF-κB regulatory complexes, indicating that anthocyanin activity is not limited to antioxidant effects but extends to receptor-mediated signaling modulation in HNSCC models.

A defining feature of anthocyanins is their very low systemic bioavailability in intact form. Following ingestion, these compounds undergo rapid metabolism, including deglycosylation and microbial transformation. As a result, circulating levels of parent anthocyanins are transient and low, and most tissues are exposed predominantly to metabolic derivatives rather than intact compounds. These downstream metabolites include smaller phenolic acids such as PCA (discussed below), along with a range of microbial catabolites that display greater chemical stability and improved systemic exposure. Accordingly, anthocyanins are best conceptualized not as directly bioactive circulating molecules, but as dietary precursor structures that expand into a metabolically active pool of compounds. This precursor-metabolite relationship represents a key structure–activity paradigm in berry phytochemistry, where the biological efficacy depends on sequential chemical transformation rather than direct receptor binding of parent anthocyanins. Within this framework, many of the biological effects attributed to anthocyanin-rich foods are more plausibly explained by the combined activity of their metabolites and host–microbiome transformation products. Although anthocyanin-containing extracts have been extensively studied in preclinical cancer models, their biological activity is best understood as modulation of redox balance and cellular signaling networks rather than highly specific molecular targeting. These compounds influence pathways associated with oxidative stress, inflammation, and cellular homeostasis, thereby contributing to suppression of tumor-promoting processes [26]. However, many of these mechanistic effects are context-dependent and are further shaped by their metabolic derivatives and tissue distribution. In the context of HNSCC, emerging studies demonstrate that anthocyanin-rich extracts can inhibit proliferation and alter cell cycle progression in oral cancer models, with additional effects on cellular differentiation and stress response pathways [28,29]. However, these outcomes are difficult to ascribe solely to intact anthocyanins, as metabolite generation occurs rapidly under physiological conditions. Anthocyanins present both opportunities and challenges to drug discovery side. Instead, anthocyanins should be viewed as structural scaffolds that generate a cascade of biologically active metabolites, which collectively engage immune, metabolic, and stress response pathways relevant to HNSCC. Their investigational potential therefore lies in their role as upstream precursors within a broader phytochemical system rather than as single active pharmacological agents. As summarized in Table 1, anthocyanins such as cyanidin-3-glucoside and delphinidin target multiple oncogenic pathways including EGFR, VEGF/VEGFR2, and NF-κB, supporting their relevance in preclinical models of HNSCC prevention and therapeutic investigation. Collectively, anthocyanins represent a central component of berry phytochemistry that contributes to biological activity through metabolic transformation rather than direct systemic action. Their structural diversity, capacity to generate bioactive derivatives, and indirect modulation of signaling networks underscore their importance in chemoprevention strategies and as lead frameworks for future multi-target therapeutic development.

### 3.2. Ellagitannins

Ellagitannins are a class of hydrolyzable polyphenols enriched in berries such as BRB, strawberries, and pomegranates, and represent a major source of bioactive metabolites relevant to immune regulation. Structurally, these compounds are characterized by HHDP groups that confer strong redox activity but also limit direct absorption due to their size and polarity [30]. As a result, their biological effects are largely mediated through metabolic transformation rather than direct systemic exposure.

Following ingestion, ellagitannins are hydrolyzed to ellagic acid and subsequently metabolized by the gut microbiota into urolithins, which exhibit improved bioavailability and systemic activity [31]. The degree of lactone ring formation and hydroxyl substitution in ellagic acid-derived metabolites strongly influences their anti-inflammatory and kinase-modulatory activity, representing a clear SAR within this class. Notably, these compounds can also reshape the microbial environment, promoting beneficial taxa such as *Akkermansia muciniphila* and *Bifidobacterium* species, thereby reinforcing anti-inflammatory signaling within the gut-immune axis [31].

Immunologically, ellagitannins and their metabolites modulate key pathways that govern inflammatory tone and immune cell function. They suppress NF-κB driven transcriptional programs and reduce pro-inflammatory cytokines such as IL-6 and TGF-β, while influencing the balance of immunosuppressive cell populations within the TME [32,33]. These effects are particularly relevant in HNSCC, where Tregs, MDSCs, and tumor-associated macrophages (TAMs) contribute to immune evasion and tumor persistence [33]. Emerging evidence suggests that ellagitannin-derived metabolites can partially reprogram this immunosuppressive landscape, linking dietary components to modulation of tumor–immune interactions [32].

In parallel, ellagitannins influence epithelial and stromal signaling pathways that intersect with immune regulation. Their metabolites have been shown to modulate cell cycle regulators, attenuate pro-survival signaling, and inhibit pathways such as Wnt signaling that contribute to tumor progression [32,34]. They also affect extracellular matrix remodeling through regulation of matrix metalloproteinases, suggesting a role in limiting invasion and shaping the TME [35]. Table 1 further highlights the mechanistic overlap between ellagic acid, resveratrol, and quercetin in regulating NF-κB, PI3K/AKT, and apoptotic pathways relevant to HNSCC progression. Collectively, ellagitannins show how dietary phytochemicals function as immunometabolic modulators rather than classical single-target agents. Their activity emerges from a combination of microbial transformation, multi-pathway signaling effects, and context-dependent interactions within the TME. These properties support the role of position ellagitannins as key contributors to the network-based mechanisms through which berry-derived compounds may influence HNSCC biology.

### 3.3. Protocatechuic Acid

Although PCA is structurally classified as a phenolic acid, it is considered separately here due to its central role as a key bioactive metabolite-derived from anthocyanins and ellagitannins and its emerging importance in mediating systemic immunometabolic effects. As a product of both host and microbial metabolism, PCA represents a critical functional link between dietary phytochemical intake and biological activity in vivo. Compared with its parent compounds, PCA exhibits greater chemical stability and bioavailability, enabling it to contribute substantially to the systemic effects associated with berry-derived interventions [8,9].

PCA has emerged as a modulator of both innate and adaptive immune responses. It influences DC activation and antigen presentation, thereby shaping downstream T cell responses and immune priming [17,18]. In addition, PCA affects the balance between effector T cells and immunosuppressive Treg populations, a key determinant of immune responsiveness within the TME [18]. These properties are particularly relevant in HNSCC, where impaired antigen presentation and expansion of suppressive immune subsets contribute to tumor progression and resistance to immunotherapy. Beyond immune regulation, PCA operates at the interface of inflammation and metabolism. It modulates redox-sensitive signaling pathways, including NF-κB, and attenuates the production of pro-inflammatory mediators, thereby limiting chronic inflammatory signaling that drives epithelial transformation [17,22]. Emerging evidence further suggests that PCA influences metabolic pathways linked to immune cell function, supporting a role in immunometabolic reprogramming within the TME. Through these combined effects, PCA contributes to the reshaping of local signaling networks toward a less tumor permissive state.

The activity of PCA highlights a broader principle in phytochemical research where metabolites generated through host–microbiome interactions often serve as the primary mediators of systemic effects. This concept is particularly relevant for berry-derived compounds, where parent molecules with limited bioavailability give rise to smaller, more bioactive intermediates capable of engaging immune and metabolic pathways. Collectively, PCA demonstrates how metabolite-driven mechanisms bridge dietary exposure and tumor immunology. By integrating effects on antigen presentation, T cell dynamics, inflammatory signaling, and metabolism, PCA functions as a central immunometabolic regulator and provides a mechanistic foundation for investigating the chemopreventive and therapeutic potential of berry-derived phytochemicals in HNSCC.

### 3.4. Flavonols

Flavonols are a widely distributed subclass of flavonoids present in berries, including compounds such as quercetin, kaempferol, and myricetin. Although typically less abundant than anthocyanins, flavonols contribute significantly to the biological activity of berry matrices due to their capacity to modulate diverse signaling pathways. Structurally, they are characterized by a hydroxylated flavone backbone, with variations in hydroxylation patterns influencing their redox properties and interaction with cellular targets [22]. The number and position of hydroxyl groups on the flavonol backbone strongly influence kinase binding affinity and ROS scavenging capacity, forming a key structure–activity determinant. In contrast to anthocyanins and ellagitannins, flavonols exhibit relatively greater stability and bioavailability, allowing them to exert both local and systemic effects. Their activity extends beyond antioxidant function and is increasingly understood in the context of signaling modulation. Flavonols interact with multiple kinases and transcription factors, including NF-κB and STAT3, thereby influencing pathways that regulate inflammation, cell survival, and immune responses [22]. Their planar C6-C3-C6 structure enables interaction with ATP-binding pockets of kinases, providing a mechanistic basis for multi-target inhibition in HNSCC.

Flavonols modulate both innate and adaptive immune processes. They regulate cytokine production, influence macrophage polarization, and modulate T cell responses, collectively contributing to the control of inflammatory tone. These effects are particularly relevant in HNSCC, where persistent inflammation and immune dysregulation shape the TME. Consistent with this, flavonols such as quercetin have been shown to inhibit proliferation and induce apoptosis in oral squamous cell carcinoma models through modulation of NF-κB, STAT3, and PI3K/Akt signaling pathways [36,37]. In addition to immune modulation, flavonols influence cellular processes associated with tumor progression, including cell cycle regulation, apoptosis, and oxidative stress responses (Table 1). Their ability to target multiple signaling nodes simultaneously aligns with the concept of network pharmacology and complements the activity of other berry-derived phytochemicals. Rather than acting as dominant drivers of specific pathways, flavonols contribute to the broader systems-level effects of berry matrices through coordinated, multi-target interactions. Collectively, flavonols represent an important class of berry-derived compounds that reinforce the immunomodulatory and anti-inflammatory properties of these dietary interventions. Their relative stability, capacity for systemic activity, and ability to engage multiple signaling pathways support their investigation as complementary components within multi-component strategies aimed at modulating tumor–immune interactions in HNSCC.

### 3.5. Phenolic Acids

Phenolic acids constitute a major class of plant-derived phytochemicals widely distributed in fruits, vegetables, whole grains, and particularly in berries such as blueberries, strawberries, raspberries, and cranberries, where they contribute to antioxidant and anti-inflammatory activity [38]. Prominent representatives include caffeic acid, ferulic acid, chlorogenic acid, and p-coumaric acid. In addition to direct dietary intake, phenolic acids are generated as major downstream metabolites of anthocyanins and other polyphenols, and often represent the principal circulating bioactive species in vivo [25,39].

Compared with structurally complex polyphenols such as anthocyanins or ellagitannins, phenolic acids are relatively small, chemically stable molecules with greater metabolic accessibility. Their low molecular weight and single aromatic ring structure enable rapid systemic absorption and broad tissue distribution, but limit target specificity compared to flavonoids and ellagitannins. This property facilitates broader tissue distribution and positions phenolic acids as key downstream effectors of berry-derived bioactivity. Notably, PCA (discussed in Section 3.3) shows the central role of these metabolites, linking anthocyanin metabolism to functional biological outcomes.

Phenolic acids such as caffeic acid and carnosic acid primarily act as modulators of cellular homeostasis rather than highly specific molecular inhibitors. They influence redox balance, inflammatory tone, and stress responsive pathways, including Nrf2 and NF-κB, and interact with signaling networks that govern proliferation and survival [25,35]. Their systemic effects are shaped by metabolic transformation, cumulative tissue exposure, and local concentrations, emphasizing the importance of considering these molecules within the broader context of berry-derived phytochemical networks.

Phenolic acids offer several advantages. Structural variation in hydroxyl and methoxy substitutions directly influences redox potential and anti-inflammatory potency, reflecting a simple but functional SAR profile. Their relatively simple chemical scaffolds, stability, and amenability to structural modification make them attractive candidates for medicinal chemistry optimization. Hydroxycinnamic acid derivatives, such as ferulic acid, have demonstrated the capacity to influence cell cycle progression, apoptosis, and angiogenic signaling in preclinical cancer models [40], supporting their potential as investigational lead compounds for anticancer development. Emerging evidence also suggests that phenolic acids may modulate the TME, including immune and metabolic pathways, although these mechanisms remain incompletely characterized and are discussed in subsequent sections. Despite promising preclinical data, clinical translation is constrained by bioavailability limitations, rapid metabolism, and interindividual variability. Advances in nanoformulation, targeted delivery strategies, and structural optimization may help overcome these barriers. Collectively, phenolic acids represent a functionally important and pharmacologically tractable class of berry-derived metabolites. By serving as bioavailable intermediates that link complex polyphenols to systemic biological effects, they underscore the potential relevance of berry phytochemicals in dietary chemoprevention research and investigational drug development strategies for cancers such as HNSCC.

### 3.6. Polyamines and Nitrogen Containing Bioactive Compounds

In addition to polyphenols, berries contain a range of nitrogen containing bioactive molecules, including polyamines such as spermine, spermidine, and putrescine, as well as structurally diverse aromatic glycosides containing polyamine fragments. These compounds have received increasing attention due to their important roles in cellular proliferation, immune regulation, and metabolic homeostasis, all of which are highly relevant to carcinogenesis and tumor progression [11,41].

Their aliphatic cationic structure enables electrostatic interactions with DNA and RNA, directly influencing chromatin architecture and gene expression. Because rapidly proliferating tumor cells exhibit increased polyamine demand, dysregulated polyamine metabolism is recognized as a hallmark of multiple cancers, including HNSCC [10,41]. In HNSCC, metabolomic profiling has revealed significant alterations in polyamine pathways, with increased levels of spermine and related metabolites associated with tumor progression and poor clinical outcome [42]. More recently, elevated expression of spermine synthase (SMS), a key enzyme responsible for spermine biosynthesis, has been shown to promote HNSCC cell proliferation, migration, and invasion, while SMS knockdown significantly suppresses tumor growth, supporting spermine metabolism as both a biomarker and a potential therapeutic target [42]. Beyond tumor-intrinsic functions, polyamines exert profound immunomodulatory effects within the TME. High extracellular spermine concentrations suppress macrophage activation, inhibit dendritic cell maturation, and impair CD8^+^ T cell effector function, thereby promoting an immunosuppressive milieu favorable for tumor progression [41,43]. Polyamine metabolism also influences macrophage polarization, MDSC activity, and Treg function, linking metabolic adaptation to immune escape and resistance to immune checkpoint blockade [10,41]. These mechanisms are particularly relevant in HNSCC, where immune suppression and defective antigen presentation are major barriers to effective immunotherapy. Conversely, controlled modulation of polyamine pathways may have therapeutic relevance. Spermidine, for example, has been shown to support autophagy, mitochondrial fitness, and immune homeostasis, illustrating the context-dependent effects of polyamine metabolism and emphasizing that therapeutic benefit may depend on restoring metabolic balance rather than indiscriminate depletion [43,44]. Pharmacologic targeting of polyamine synthesis and transport is therefore being explored as a strategy to enhance antitumor immunity and improve responsiveness to PD-1/PD-L1 blockade. In addition to free polyamines, spermine-containing aromatic glycosides and related nitrogenous metabolites identified in berries may represent underexplored lead structures for anticancer drug discovery. Their chemical versatility and capacity to engage multiple signaling pathways suggest potential relevance in both immunometabolic regulation and chemoprevention. Although these compounds remain less studied than berry polyphenols, their emerging relevance suggests that future research should expand beyond traditional flavonoid centered models to include nitrogen containing metabolites as integral contributors to berry-mediated chemoprevention and investigational therapeutic development.

Taken together, the diverse phytochemical classes present in berries including anthocyanins, ellagitannins, PCA, flavonols, phenolic acids, and the less explored polyamines and other nitrogen containing bioactive compounds converge on overlapping molecular and cellular pathways that are central to HNSCC pathogenesis. While polyphenols primarily regulate inflammatory signaling, oxidative stress, immune responses, and metabolic reprogramming, polyamines and related nitrogenous metabolites further contribute through modulation of cellular proliferation, chromatin dynamics, immune suppression, and tumor metabolic adaptation. A consolidated overview of berry-derived compounds structural features, metabolic transformation, and major biological targets is provided in Table 1. Integrating these individual profiles highlights how berry-derived compounds function not as isolated agents, but as coordinated multi-component modulators of interconnected oncogenic, immunologic, and metabolic networks, providing a systems-level framework for investigating their chemopreventive and therapeutic potential. These compound-specific molecular effects summarized in Table 1 provide the mechanistic basis for the broader immunomodulatory and metabolic reprogramming effects discussed in subsequent sections.

**Table 1:** Berry-derived phytochemicals: molecular characterization and HNSCC-specific pharmacological evidence: Summary of major berry-derived phytochemicals, including stilbenes, flavonoids, ellagitannins, anthocyanins, condensed tannin, and polyphenolic metabolites with their principal berry sources, validated molecular targets, signaling pathways, experimental evidence, and therapeutic relevance in HNSCC. The table highlights key oncogenic pathways modulated by these compounds, including EGFR, PI3K/AKT, NF-κB, STAT3, MAPK, Wnt/β-catenin, and immunoregulatory pathways, together with their verified effects in HNSCC cell lines, animal models, and/or preclinical/early clinical studies. Synergistic interactions with conventional therapies such as cisplatin, cetuximab, and immunomodulatory agents are also included, emphasizing their translational potential for chemoprevention and adjunctive therapy.

Compound (Trivial Name)	IUPAC Name	Molecular Formula	MW (g/mol)	PubChem CID	Chemical Family	Berry Sources	Key HNSCC Targets & Pathways	Evidence Context	Effect in HNSCC (Cell Lines/Models)	Synergistic Interactions	Refs.
Resveratrol	5-[(1E)-2-(4-hydroxyphenyl)ethenyl]benzene-1,3-diol	C_14_H_12_O_3_	228.24	445154	Stilbene	Grape skin, Blueberry, Cranberry, Mulberry	NF-κB, PI3K/Akt, MAPK, MMP-2/9, p53/PTEN, AP-2, CBX7/p16, Smad4, caspase-3/9	Preclinical/Early clinical	Antiproliferative, antimetastatic, proapoptotic in OSCC lines (SCC-9, SCC-25, Cal27). G2/M and G1-S arrest; caspase-3/9 activation; EMT suppression via Smad4. 60–70% reduction of oral preneoplastic lesion onset in hamster buccal pouch (DMBA) model. Selective DNA damage in HNSCC vs. normal keratinocytes. IC_50_ 10–100 µM; dose-dependent effects confirmed in vivo.	Cisplatin: ↑ p21, ↑cytotoxicity in HNSCC lines. EGCG: ↑ apoptosis, ↓ tumor volume in HNC in vivo. Curcumin: ↑ bioavailability. Ellagic acid: synergistic caspase-3 induction (CI = 0.64, isobolographic).	[45,46,47]
Quercetin	2-(3,4-dihydroxyphenyl)-3,5,7-trihydroxy-4H-chromen-4-one	C_15_H_10_O_7_	302.24	5280343	Flavonol	Cranberry, Blueberry, Blackcurrant, Elderberry, Lingonberry	EGFR/Akt/FOXO1, MMP-2/9, JAK/STAT3, PI3K/NF-κB, caspase-3	Preclinical/Early clinical	10 µM suppresses migration/invasion in EGFR-overexpressing HSC-3 and FaDu cells; inhibits MMP-2/9 protein expression and proteolytic activity; blocks 3D colony formation in Matrigel. FOXO1-mediated G1 arrest in EGFR-overexpressing oral SCC. Inhibits SAS oral cancer invasion via NF-κB/MMP-2/9 axis. IC_50_ 5–50 µM.	Resveratrol: synergistic caspase-3 (CI = 0.68). Phytonutrient mix (quercetin + resveratrol + curcumin + EGCG): 67.6% growth inhibition and 63.6% tumor burden reduction in FA-HNSCC mouse model.	[38,48,49]
EGCG (Epigallocatechin-3-gallate)	[(2R,3R)-5,7-dihydroxy-2-(3,4,5-trihydroxyphenyl)-3,4-dihydro-2H-chromen-3-yl] 3,4,5-trihydroxybenzoate	C_22_H_18_O_11_	458.37	65064	Flavan-3-ol	Blackberry, Raspberry, Strawberry, Gooseberry	EGFR/HER2, AKT/STAT3, mTOR, MDR1 (P-gp), DNMT, Notch1/2, LC3B/Beclin-1 (autophagy)	Preclinical/Early clinical	G1 arrest in HSC-3 cells; inhibits DNA synthesis and proliferation in SCC-25. MDR1 downregulation reverses cisplatin resistance in oral CAR cells via AKT/STAT3 suppression. Autophagy and caspase-3/9 activation confirmed. IC_50_ 20–80 µM across OSCC lines; 40–70% reduction in migration/invasion in scratch and Transwell assays.	Cisplatin: MDR1/AKT suppression → re-sensitization. Resveratrol: ↑ apoptosis + ↓ tumor volume in HNC in vivo. Luteolin: ↑ paclitaxel cytotoxicity in HNSCC. Curcumin: synergistic G2/M arrest.	[50,51,52]
Ellagic acid	2,3,7,8-tetrahydroxy-chromeno [5,4,3-cde]chromene-5,10-dione	C_14_H_6_O_8_	302.19	5281855	Ellagitannin	Raspberry, Strawberry, Blackberry, Pomegranate	NF-κB, Wnt/β-catenin, CYP1A1/1B1, caspase-3/7, G0/G1 checkpoint, carcinogen-DNA adduct formation	Early clinical/Preclinical	Selectively cytotoxic to HSC-2 oral carcinoma cells; caspase-3/7 activation with PARP cleavage. Suppresses Wnt/β-catenin and NF-κB in DMBA-induced hamster buccal pouch carcinoma model. Direct carcinogen-DNA adduct inhibition relevant to tobacco-driven HNSCC. IC_50_ 10–100 µM in vitro.	Resveratrol: synergistic caspase-3 induction (CI = 0.64, isobolographic). Quercetin: additive apoptosis. Gut microbiota converts ellagic acid → Urolithin A (secondary anti-inflammatory effects).	[53,54,55]
Cyanidin-3-glucoside (C3G)	2-(3,4-dihydroxyphenyl)-5,7-dihydroxy-3- -D-glucopyranosyloxychromenylium	C_21_H_21_O_11_^+^	449.39	197081	Anthocyanin	Blueberry, Blackcurrant, Elderberry, Bilberry, Chokeberry	NF-κB, VEGF/VEGFR2, MRP-1, NADPH oxidase, eNOS, caspase-1/NLRP3 (pyroptosis), IL-1β	Early translational evidence	Induces pyroptosis in OSCC cells via caspase-1/NLRP3/IL-1β axis. Berry extract-derived anthocyanins inhibit MRP-1 in endothelial tumor cells → GSSG nuclear accumulation → apoptotic death. Anti-angiogenic via VEGF/VEGFR2 suppression. Epidemiological association with reduced oral cancer risk. Effective concentrations: 10–100 µM.	Cisplatin: nephroprotective in vivo (↑ tolerable dose). Cyclophosphamide: hepatoprotective synergy (blackberry anthocyanins, mice). Delphinidin: additive NF-κB/VEGF suppression.	[29,56,57]
Delphinidin	3,3′,4′,5,5′,7-hexahydroxyflavylium	C_15_H_11_O_7_^+^	303.24	128853	Anthocyanin	Blueberry, Blackcurrant, Pomegranate, Bilberry	EGFR (competitive inhibitor), VEGFR2, ErbB2/IGF1R, PI3K/Akt/MAPK, HIF-1α, caspase-3/9, cyclin D1/PCNA	Preclinical	Inhibits EGFR- and VEGFR2-associated signaling in RTK-overexpressing cancer cells (NCI-H441, SK-MES-1). Suppresses PI3K/Akt and MAPK phosphorylation; reduces Ki-67/PCNA and CD31/VEGF in NSCLC xenograft. VEGF-induced proliferation blockade via VEGFR2 downregulation; HIF-1α suppression. Vicinal-OH B-ring structure confirmed as key pharmacophore for EGFR inhibitory potency. IC_50_ 10–50 µM.	Cetuximab: additive EGFR blockade (different binding epitope). Anti-VEGF agents: complementary anti-angiogenic axis. C3G: additive NF-κB/VEGF suppression.	[58,59,60]
Black raspberry anthocyanin extract (BRB-ACN)	Complex mixture: cyanidin-3-rutinoside, cyanidin-3-xylosylrutinoside, cyanidin-3-glucoside (predominant)	Mixture	-	-	Anthocyanin mixture	Black raspberry (*Rubus occidentalis*)	NF-κB, COX-2, EGFR, AMPK, inflammatory cytokines (IL-6, TNF-α), oxidative stress (Nrf2/HO-1), STAT3	Preclinical/Early clinical	Suppresses oral carcinogenesis and dysplasia progression in hamster buccal pouch (DMBA) and rat 4NQO oral cancer models. Mucoadhesive gel formulation (10% BRB) reduces Ki-67, COX-2, and VEGF in Phase I/II human oral dysplasia trials. Modulates inflammatory and metabolic pathways; reduces proliferation, angiogenesis, and pro-inflammatory biomarkers in OPL biopsies.	Standard chemoprevention agents: potential additive chemoprevention. 5-FU: AMPK-mediated sensitization in preclinical HNSCC models.	[15,20,61,62,63]
Procyanidins (Proanthocyanidins, PAC)	Oligomeric/polymeric flavan-3-ol units linked by C4 → C8 or C4 → C6 bonds (e.g., procyanidin B2: epicatechin dimer)	Variable (B2: C_30_H_26_O_12_)	Variable (B2: 578.52)	B2: 5320711	Condensed tannin	Cranberry, Blueberry, Chokeberry	MMP-2/9, VEGF, EGFR, ROS/oxidative stress, NF-κB, E-cadherin restoration	Preclinical	Cranberry PAC suppresses invasion (↓ MMP-2/9) and angiogenesis (↓ VEGF) in OSCC-3 and SCC-9 cells. Inhibits EGFR phosphorylation and downstream Akt signaling. Restores E-cadherin expression, reversing EMT phenotype. Reduces extracellular matrix degradation associated with OSCC progression. IC_50_ 25–100 µg/mL.	EGCG: synergistic ROS generation and apoptosis in oral cancer cells. Anthocyanins: additive NF-κB suppression. Cisplatin: ↑ cytotoxicity via EGFR co-suppression (preliminary data).	[64,65]
Urolithin A (ellagitannin gut metabolite)	3,8-dihydroxybenzo[c]chromen-6-one	C_13_H_8_O_4_	228.20	5488186	Polyphenolic metabolite	Derived from ellagic acid-rich berries (raspberry, strawberry, pomegranate) via gut microbiota biotransformation	NF-κB, autophagy (mTOR/LC3), mitochondrial biogenesis (PGC-1α), caspase-3/9, IL-6/TNF-α	Preclinical	Anti-inflammatory and antiproliferative in oral cancer-associated inflammatory microenvironments (CAL27, SCC-4 lines). Modulates mitochondrial homeostasis via PGC-1α/autophagy axis; caspase-3/9-mediated apoptosis. Bioavailability superior to parent ellagic acid. Emerging clinical safety data in Phase I trials (colorectal). IC_50_ 15–60 µM in OSCC lines.	Ellagic acid: potentiates parent compound chemoprevention (prodrug relationship). Rapamycin: additive mTOR/autophagy suppression. Cisplatin: ↓ inflammatory TME → improved drug penetration (preclinical).	[66,67]

Abbreviations: CI, combination index; DMBA, 7,12-dimethylbenzanthracene; EGCG, epigallocatechin-3-gallate; EMT, epithelial-mesenchymal transition; HNSCC, head and neck squamous cell carcinoma; IC_50_, half-maximal inhibitory concentration; MDR1, multidrug resistance protein 1; mTOR, mammalian target of rapamycin; MMP, matrix metalloproteinase; MW, molecular weight; NF-κB, nuclear factor kappa-light-chain-enhancer of activated B cells; OSCC, oral squamous cell carcinoma; OPL, oral potentially malignant lesion; PAC, proanthocyanidins; PubChem CID, PubChem Compound Identifier; RTK, receptor tyrosine kinase; TME, tumor microenvironment; VEGFR2, vascular endothelial growth factor receptor 2; Evidence levels: early = first-in-human safety data; Preclinical = in vitro and/or xenograft evidence only; ↑ = increased/upregulation/activation; ↓ = decrease/downregulation/inhibition/suppression; → = leads to/results in/converts to.

## 4. Immunomodulatory Mechanisms of Natural Compounds

**Figure 1:**
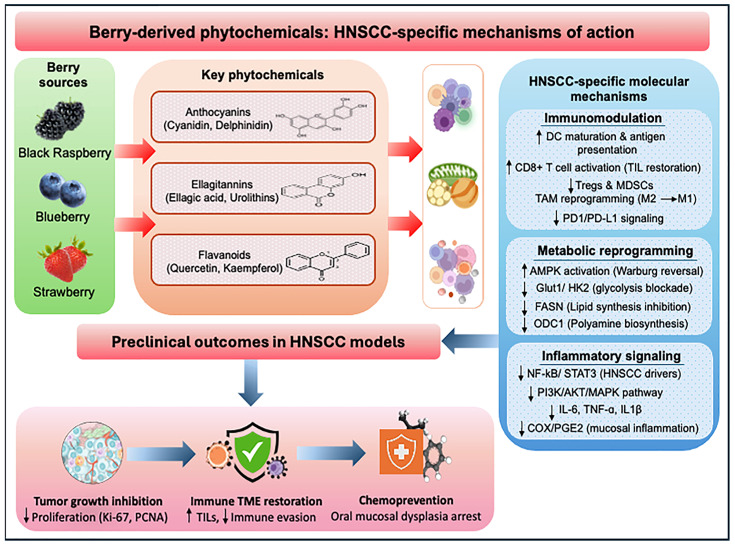
Berry-derived phytochemicals in HNSCC: HNSCC-specific mechanisms of action of berry-derived phytochemicals, encompassing immunomodulation of the TME, metabolic reprogramming of the Warburg phenotype, and suppression of inflammatory signaling pathways relevant to head and neck carcinogenesis. HNSCC = head and neck squamous cell carcinoma; TME = tumor microenvironment; TIL = tumor-infiltrating lymphocyte; ↑ = increased/upregulation/activation; ↓ = decrease/downregulation/inhibition/suppression; → = leads to/results in/converts to.

We next discuss how berry-derived metabolites influence the immune axis of the TME, with emphasis on HNSCC-relevant immune populations [17,19]. In HNSCC, which is characterized by profound immune dysregulation, modulation of immune cell function represents an important area of therapeutic investigation [15,20]. Emerging evidence suggests that phytochemicals-derived from dietary sources can enhance antitumor immunity by regulating DC activation, macrophage polarization, Tregs function, and inflammatory signaling pathways, thereby potentially improving immune surveillance and therapeutic responsiveness [21,68]. These immunomodulatory mechanisms do not occur independently but function as an interconnected network within the HNSCC TME. Natural compounds, particularly berry-derived phytochemicals, simultaneously influence antigen presentation by DC, macrophage polarization, Tregs expansion, and cytokine-driven inflammatory signaling. These coordinated effects may collectively shift the TME from an immunosuppressive, tumor-promoting state toward enhanced immune surveillance and antitumor immunity. Rather than acting through a single pathway, these compounds exert systems-level immune reprogramming that may influence therapeutic responsiveness, including sensitivity to immune checkpoint blockade in preclinical settings. Moreover, these interconnected immunomodulatory mechanisms do not function independently but collectively shape the HNSCC TME, as summarized in Figure 1.

### 4.1. Dendritic Cell Activation and Antigen Presentation

Dendritic cells are pivotal antigen presenting cells that integrate environmental signals to orchestrate adaptive immunity. Upon activation, DCs undergo maturation marked by upregulation of co-stimulatory molecules (CD80, CD86) and major histocompatibility complex class II, and secrete cytokines such as IL-12 that guide effector T cell differentiation [69,70]. In tumors, DC function is frequently impaired, resulting in suboptimal T cell priming and immune evasion.

Accumulating evidence indicates that dietary phytochemicals, particularly berry-derived compounds, can modulate DC function and influence the tumor–immune microenvironment. BRB-derived metabolites, including PCA and other anthocyanin-derived products, attenuate DC activation in inflammatory contexts. In a murine contact hypersensitivity model, dietary BRB and PCA reduced DC accumulation and downregulated co-stimulatory molecules such as CD80, indicative of impaired DC maturation and antigen presenting capacity [17,71]. These interventions also reduced DC trafficking to draining lymph nodes, suggesting dampened initiation of adaptive immune responses. Mechanistically, these phytochemicals converge on signaling pathways central to DC activation. Inhibition of ERK phosphorylation and downregulation of activation markers (CD40, CD80) are accompanied by suppressed secretion of pro-inflammatory cytokines, including IL-6 and IL-12, which are critical for T cell priming [72]. Consequently, antigen specific T cell responses, including proliferation and production of IFN-γ and IL-17, are reduced, reflecting attenuated effector T cell activation [18]. In human DCs, PCA suppresses pro-inflammatory cytokines such as IL-6, IL-8, and CCL2 while impairing chemokine-directed migration, thereby limiting DC capacity to reach lymph nodes and initiate antigen specific responses. These effects are mediated in part through PPAR-γ, linking metabolic signaling to immune regulation [73] and align with emerging observations in HNSCC, where impaired DC maturation and antigen presentation contribute to immune evasion [74]. However, direct validation of berry-derived phytochemicals on DC function in HNSCC-specific models remains limited, highlighting an important area for further investigation. Chronic DC activation and sustained cytokine production contribute to tumor-promoting inflammation and immune dysfunction [75]. In HNSCC, characterized by immune dysregulation and chronic inflammation, modulation of DC maturation, cytokine secretion, and T cell priming by dietary phytochemicals may help restore immune balance and limit pro-tumorigenic inflammation (Figure 1). Collectively, these observations support a model in which berry-derived phytochemicals fine-tune DC activation states rather than indiscriminately suppress immune function. As illustrated in Figure 1, by modulating antigen presentation and T cell priming, DCs emerge as central targets for natural compounds in shaping antitumor immunity, highlighting their investigational potential utility in preventive and adjunctive cancer strategies.

Because DC-mediated antigen presentation shapes subsequent immune cell recruitment and activation, alterations in DC function are closely connected to macrophage polarization and broader myeloid reprogramming within the TME. The following section therefore examines how natural compounds influence macrophage phenotypes and tumor-associated myeloid responses, further contributing to immune restoration in HNSCC.

### 4.2. Macrophage Polarization and Myeloid Reprogramming

Macrophages are highly plastic immune cells whose phenotype is shaped by microenvironmental cues. In tumors, macrophages adopt distinct polarization states broadly classified as pro-inflammatory M1 or anti-inflammatory M2 phenotypes. M1 macrophages produce pro-inflammatory cytokines, including IL-12 and TNF-α, and exhibit potent tumoricidal activity, whereas M2 macrophages secrete immunosuppressive mediators such as IL-10 and TGF-β and promote tissue remodeling, angiogenesis, and tumor progression [76,77]. Figure 1 further highlights how these effects extend beyond DC regulation to include macrophage polarization and broader myeloid reprogramming within the TME. Within the TME, macrophages are often skewed toward an M2-like state, referred to as TAMs, which contribute to immune evasion, suppression of cytotoxic T cell responses, and metastasis [78]. TAM abundance correlates with poor prognosis in HNSCC and other solid tumors, underscoring their role as central mediators of immunosuppression [71]. Emerging studies indicate that natural compounds, particularly berry-derived phytochemicals such as metabolites originating from anthocyanins and ellagitannins, can modulate macrophage polarization and reverse tumor-promoting myeloid programs. Preclinical evidence demonstrates that dietary BRB extracts and PCA shift macrophage phenotypes toward M1-like profiles, enhancing expression of pro-inflammatory cytokines (IL-12, TNF-α) while reducing M2-associated markers, including IL-10, arginase-1, and CD206 [19,79]. This reprogramming is associated with enhanced antitumor immune responses in preclinical models by increasing cytotoxic T cell recruitment and activity, highlighting the central role of macrophages as intermediaries through which dietary compounds shape the immune landscape. Mechanistically, berry-derived metabolites target signaling pathways that govern macrophage polarization, including NF-κB, STAT3, and PI3K/AKT. Inhibition of STAT3 and NF-κB signaling reduces M2-associated immunosuppressive cytokine production, whereas enhanced NF-κB activation in M1 macrophages promotes pro-inflammatory and tumoricidal activity [39,40]. These effects demonstrate that natural compounds can finely tune macrophage functional states rather than globally activating or suppressing macrophages, thus potentially mitigating systemic inflammation while supporting restoration of antitumor immunity. Beyond macrophages, BRB phytochemicals also influence other myeloid populations, including MDSCs, by reducing their accumulation and immunosuppressive activity in the TME [80]. While these findings are supported by preclinical and mechanistic studies, direct evidence in HNSCC-specific models remains relatively limited. However, the well-established role of TAM driven immunosuppression and myeloid dysregulation in HNSCC supports the biological relevance of these mechanisms in this disease context [81]. Further studies are needed to determine whether berry-derived phytochemicals can reproducibly modulate macrophage polarization and myeloid function in HNSCC patients. Together, these findings support a model in which natural compounds remodel the myeloid compartment to favor antitumor immunity. Although several mechanistic insights are derived from non-HNSCC tumor systems, they highlight conserved pathways that are also implicated in HNSCC biology, supporting TAM reprogramming as a promising area for cancer prevention and adjunctive therapeutic investigation, particularly in inflammation-driven malignancies such as HNSCC. The coordinated regulation of TAM polarization, MDSC suppression, and cytotoxic T cell recruitment is summarized in Figure 1, emphasizing macrophages as central mediators of berry-driven immune remodeling.

In parallel with macrophage reprogramming, suppression of regulatory immune populations such as Tregs further contributes to the restoration of effective antitumor immunity. The following section focuses on how natural compounds modulate Treg expansion and function, thereby reducing immunosuppression and enhancing cytotoxic T cell responses within the HNSCC TME.

### 4.3. Regulatory T Cell Modulation and Immune Suppression

Tregs are central mediators of immune tolerance, characterized by expression of FoxP3 and the high affinity IL-2 receptor CD25 [82]. Within the TME, Tregs suppress antitumor immunity by inhibiting CD4^+^ helper and CD8^+^ cytotoxic T cell activation, thereby limiting responses against tumor-associated antigens [83]. Immunosuppressive mechanisms employed by Tregs include secretion of inhibitory cytokines, upregulation of checkpoint molecules such as CTLA-4, and consumption of IL-2 via high CD25 expression [84,85]. Elevated Treg infiltration is associated with poor prognosis in HNSCC patients, reflecting their contribution to an immunosuppressive TME [86]. Recent integrative analyses of tumor-infiltrating immune cells in HNSCC further highlight that the balance between effector T cells and suppressive populations, including Tregs and myeloid cells, critically determines responsiveness to immunotherapy [87].

Emerging evidence demonstrates that dietary phytochemicals, particularly those derived from berries, can modulate Treg activity and reshape the tumor–immune landscape. In preclinical HNSCC models, BRB-derived extracts reduce tumor-infiltrating CD4^+^FoxP3^+^ Tregs, accompanied by downregulation of FoxP3, CD25, and CTLA-4 expression [19,79]. These effects are associated with enhanced antitumor immunity in preclinical models, including increased infiltration of CD8^+^ cytotoxic T cells and elevated granzyme B and perforin expression, suggesting improved effector T cell function. Mechanistically, BRB-derived phytochemicals modulate pathways critical for Treg expansion and suppressive activity. For example, inhibition of IL-2 induced STAT5 phosphorylation limits Treg proliferation and survival, while concomitant reductions in MDSCs accumulation further relieve immunosuppressive pressure within the TME [80,88]. Similar effects have been observed in UVB-induced cutaneous carcinogenesis models, where topical BRB extracts reduced tumor burden and Treg infiltration [89]. Although these findings originate from non-HNSCC models, they highlight conserved immunoregulatory pathways that might also be implicated in HNSCC, particularly those governing Treg expansion and immune suppression. However, direct validation of these mechanisms in HNSCC-specific systems remains limited. In parallel, BRB interventions decrease PD-L1 expression on myeloid populations while upregulating co-stimulatory molecules such as CD86, indicating broader remodeling of immune regulatory networks [19].

Beyond modulating Tregs, berry-derived phytochemicals may also influence responsiveness to immunotherapy. Extracts from *Rubus coreanus*, a species of berry, have been reported to block the PD-1/PD-L1 interaction in vitro and suppress tumor growth in humanized PD-1 mouse models [90]. Collectively, these studies suggest that dietary berries and their phytochemicals reprogram the tumor–immune microenvironment by suppressing Treg-mediated immunosuppression, limiting additional inhibitory populations, and promoting cytotoxic T cell activity. This integrated regulation of Tregs, checkpoint signaling, and effector T cell activation is represented in Figure 1 as a major mechanism underlying improved immune surveillance. While several of these findings are derived from preclinical or non-HNSCC tumor models, they are consistent with the established role of Tregs in driving immune evasion in HNSCC [91]. Further HNSCC-specific studies are required to confirm the extent to which these mechanisms are conserved and relevant for therapeutic investigation.

Beyond the direct regulation of Treg-mediated immune suppression, effective antitumor immunity also depends on broader control of cytokine networks and chronic inflammatory signaling that sustain tumor progression. The following section therefore focuses on how berry-derived phytochemicals, modulate inflammatory cytokines and key signaling pathways such as NF-κB, STAT3, PI3K/AKT, and MAPK/ERK to restore immune balance and limit pro-tumorigenic inflammation in HNSCC.

### 4.4. Cytokine and Inflammatory Signaling Modulation

Chronic inflammation is a central driver of HNSCC initiation and progression, shaping a tumor permissive microenvironment through sustained activation of inflammatory signaling pathways and dysregulated cytokine production [75,92]. Key molecular mediators of this process include NF-κB, STAT3, PI3K/AKT, and MAPK/ERK, which orchestrate the transcription of pro-inflammatory cytokines (IL-6, TNF-α, IL-1β), chemokines, COX-2, and anti-apoptotic proteins, collectively promoting tumor cell proliferation, immune evasion, and resistance to apoptosis [93,94]. Therefore, attenuation of these cytokines and upstream signaling pathways represents a biologically relevant mechanism for cancer prevention and therapeutic investigation. Immune evasion in HNSCC is further reinforced by cancer-associated fibroblasts, immune checkpoint activation, and TP53-associated remodeling of the TME, which collectively promote resistance to immune-mediated clearance [95]. Dietary berries and their bioactive phytochemicals have emerged as potent modulators of inflammatory signaling in cancer relevant contexts. In a 4NQO-induced rat model of oral carcinogenesis, administration of freeze-dried BRBs or their constituent ellagic acid significantly reduced oral lesion incidence and multiplicity by ~40%, concomitant with suppression of pro-inflammatory and survival pathways [19]. BRB and ellagic acid treatments downregulated mRNA expression of key inflammatory biomarkers, including *Cxcl1*, *Mif*, *Nfe2l2*, *IL-1β*, *NF-κB*, and *Ptgs2*, while concurrently reducing expression of genes involved in cell cycle progression and anti-apoptotic signaling (*Birc5*, *Aurka*, *Ccna1*, *Ccna2*). These findings indicate that BRB-derived phytochemicals disrupt the interplay between chronic inflammation, oxidative stress, and cell survival that underlies tumorigenesis.

Importantly, limited early translational clinical studies have reported findings consistent with these preclinical observations. Oral administration of BRB troches to patients with oral squamous cell carcinoma led to decreased expression of pro-survival genes (*Aurka*, *Birc5*, *Egfr*) and pro-inflammatory mediators (*Nfkb1*, *Ptgs2*), supporting the translational relevance of dietary modulation of inflammatory signaling [96]. Mechanistically, BRB treatment also restored local GC homeostasis by upregulating 11β-hydroxysteroid dehydrogenase type 2 (HSD11β2) expression and reducing active corticosterone levels, highlighting a novel pathway by which dietary compounds may help to modulate GC-driven inflammatory signaling during oral carcinogenesis [21]. The anti-inflammatory properties of berries extend beyond BRB. Further, dietary strawberries attenuated colorectal tumorigenesis by reducing phosphorylation of PI3K, AKT, ERK, and NF-κB, and decreasing TNF-α, IL-1β, IL-6, iNOS, and COX-2 levels [97]. Mulberry extracts similarly suppressed NF-κB/p65 and ERK/MAPK activation, reducing IL-1β, IL-6, iNOS, and COX-2 expression in LPS-stimulated macrophages [98]. In esophageal carcinogenesis models, BRB decreased COX-2, iNOS, c-Jun, and glandin E2 levels while inhibiting angiogenesis, IL-1β, and IL-12 production, and reducing macrophage and neutrophil infiltration [99,100,101]. Additional studies showed that multiple berry types, including BRBs, red raspberries, strawberries, blueberries, and wolfberries, lower circulating pro-inflammatory cytokines such as IL-5 and GRO/KC, highlighting their broad anti-inflammatory efficacy [102]. Although much of the available evidence originates from non-HNSCC systems, the signaling pathways involved are highly conserved and are known to play central roles in HNSCC pathogenesis [103,104]. Nevertheless, the extent to which these effects translate directly to HNSCC remains incompletely defined, and more disease specific mechanistic studies are still needed.

Berry-derived anthocyanins, primarily through their metabolic derivatives, and other polyphenolics also exert direct anti-inflammatory effects on immune cells. In vitro and in vivo studies demonstrate suppression of iNOS, COX-2, IL-1β, IL-6, TNF-α, IL-10, PGE2, and NF-κB signaling in macrophages and colitis models [14,105]. While these findings reinforce the broader immunoregulatory potential of berry compounds, their direct relevance to HNSCC biology has yet to be fully established. Collectively, these findings support a unified model in which berry-derived phytochemicals reshape the HNSCC immune landscape through coordinated regulation of innate and adaptive immunity. By improving DC function, promoting macrophage reprogramming toward antitumor phenotypes, limiting Treg-mediated suppression, and suppressing chronic inflammatory signaling, these compounds may help in restoring immune balance within the TME. This multi-target immunomodulatory activity may be particularly important in HNSCC, where resistance to immunotherapy is often driven by simultaneous dysfunction across several immune compartments rather than a single dominant pathway. The systems-level organization of berry-derived phytochemicals and their coordinated effects on immune regulation, metabolic reprogramming, and inflammatory signaling within the HNSCC TME is comprehensively summarized in Figure 1. These properties support continued investigation of berry-derived compounds as dietary chemopreventive agents in HNSCC and other inflammation-associated malignancies.

## 5. Immunometabolism and Metabolic Reprogramming in HNSCC and Beyond

As discussed in the preceding section, immune dysfunction in HNSCC is not solely governed by cytokine signaling or checkpoint receptor engagement but is fundamentally shaped by cellular metabolic states. Immune cell fate, activation, and persistence are tightly coupled to metabolic programming, positioning metabolism as an important regulator of antitumor immunity. In this context, berry-derived compounds may influence key metabolic features of the TME that influence tumor–immune competition. The emerging field of immunometabolism has established that glycolytic flux, mitochondrial fitness, AMPK signaling, and metabolite accumulation such as lactate accumulation influence whether immune cells sustain antitumor activity or adopt suppressive phenotypes. In HNSCC, where metabolic heterogeneity and immune evasion are prominent, metabolic reprogramming represents a central axis linking tumor progression with immune dysfunction [103].

Natural compounds, including berry-derived polyphenols, are increasingly recognized as modulators of these metabolic circuits. Beyond their established antioxidants or anti-inflammatory roles, these compounds influence glycolysis, energy sensing pathways, and metabolite accumulation within TME (Figure 1). Importantly, evidence supporting these effects spans multiple tumor types and experimental systems, underscoring the conserved nature of metabolic targeting by dietary phytochemicals.

### 5.1. Glycolysis Modulation: Rebalancing the Tumor–Immune Metabolic Axis

Aerobic glycolysis (the Warburg effect) enables tumor cells to rapidly generate biosynthetic intermediates while producing lactate as a byproduct [106]. However, glycolysis is also essential for effector T cell activation. Thus, excessive tumor glycolysis creates metabolic competition within the TME, depriving cytotoxic T cells and promoting immune exhaustion [107].

In a 4NQO-induced murine model of oral carcinogenesis (C57BL/6 mice), dietary BRB supplementation significantly altered glycolytic intermediates and reduced metabolic signatures associated with tumor progression, as determined by LC-MS-based metabolomics and pathway enrichment analysis [9]. These metabolic changes were accompanied by reduced tumor burden and modulation of immune responses in related studies [19], suggesting that attenuation of tumor-associated glycolytic dominance may influence the metabolic environment encountered by infiltrating immune cells. Supportive evidence from other experimental systems indicates that berry-derived polyphenols can influence glucose metabolism through conserved molecular pathways. For example, in human ovarian cancer cell lines (e.g., PA-1, OVCAR3, MDAH2774, and SKOV3), resveratrol which is found in berries reduced glucose uptake and interrupted GLUT1 translocation, as assessed by glucose transport assays [108]. Blueberry anthocyanins-rich extracts and their metabolites inhibited hypoxia-induced glycolytic gene expression in HT-29 colon cancer cells, accompanied via reduction of HIF-1α activity [109]. Although these findings arise from non-HNSCC models, the underlying pathways particularly HIF-1α signaling, GLUT1-mediated glucose transport, and glycolytic reprogramming are highly conserved and have also been implicated in metabolic adaptation in HNSCC under hypoxic and inflammatory conditions [110]. Nevertheless, direct mechanistic evidence linking berry-derived phytochemicals to glycolytic regulation and immune-metabolic coupling specifically in HNSCC remains limited, highlighting an important area for future investigation. Collectively, these studies, accompanied with cell-based and in vivo evidence, indicate that berry-derived polyphenols can attenuate glycolytic reprogramming across malignancies, thereby alleviating metabolic competition within the TME.

### 5.2. AMPK Activation: A Convergent Energy Sensing Mechanism

AMPK serves as a master regulator of cellular energy homeostasis, restraining anabolic growth through inhibition of mammalian target of rapamycin (mTOR) and promoting catabolic processes [111]. In immune cells, AMPK supports memory T cell differentiation and modulates macrophage polarization, and enhances metabolic resilience, thereby linking energy sensing to immune persistence and inflammatory tone [107,111]. Within HNSCC, metabolic stress, hypoxia, and nutrient deprivation frequently converge on AMPK signaling, positioning it as a key regulatory node at the interface of tumor metabolism and immune adaptation [110]. In this setting, AMPK activity may influence not only tumor cell growth but also the functional fitness of infiltrating immune populations.

Metabolomic profiling in the 4NQO oral carcinogenesis model revealed pathway signatures consistent with AMPK activation following BRB supplementation [20]. Although phosphorylation of AMPK was not measured, the observed shifts in AMP/ATP-associated metabolites are consistent with engagement of AMPK regulated pathways and suggests a metabolic environment less favorable for tumor growth. Direct biochemical from other systems further this mechanism. Evidence from additional experimental systems further supports AMPK as a conserved target of berry-derived phytochemicals across cancer types. Resveratrol induces LKB1-dependent AMPK phosphorylation in hepatocellular carcinoma and breast cancer cell lines, resulting in mTOR suppression and reduced proliferation [112]. Berry-derived anthocyanidins similarly activate AMPKα1 in colon cancer cells (HT-29), with downstream inhibition of mTOR/Akt signaling, promoting apoptosis [113]. While these findings derive from non-HNSCC models, the AMPK-mTORC1 axis is directly relevant to HNSCC: mTORC1 hyperactivation is documented in >60% of HNSCC specimens downstream of PIK3CA/PTEN alterations, and mTOR inhibition restores memory CD8^+^ T cell differentiation in immunosuppressed TME contexts. Berry-mediated AMPK activation thus offers a mechanism to simultaneously suppress tumor anabolic growth and restore immune competence through a shared regulatory node. However, direct experimental validation of berry-derived phytochemicals on AMPK signaling within HNSCC-specific tumor and immune compartments remains limited. These findings support the possibility that AMPK represents a conserved mechanistic node through which berry polyphenols influence tumor metabolism.

### 5.3. Metabolic Rewiring of the Tumor Microenvironment

Beyond tumor-intrinsic metabolism, the TME exhibits dynamic metabolic crosstalk between malignant cells, stromal populations, and infiltrating immune cells. Hypoxia-induced stabilization of HIF-1α promotes glycolysis and immunosuppressive signaling, while MDSCs and TAMs adopt metabolic programs that reinforce immune suppression [114]. In HNSCC, these coordinated metabolic and immune alterations are increasingly recognized as key determinants of immune evasion and therapeutic resistance [103,110]. Berry-derived compounds have been reported to influence these processes at both metabolic and immunologic levels. BRB extracts suppress DC activation and antigen specific T cell responses in murine inflammatory models, as evidenced by reduced CD80/CD86 expression and diminished cytokine production [18]. In oral carcinogenesis models, BRB supplementation reduced Tregs activity and inflammatory biomarkers [19]. A recent study suggest that dietary polyphenols may indirectly influence immune activation by altering stromal metabolic outputs, including lactate and arginine availability within the TME [115]. While these studies primarily report immunologic endpoints, integration with metabolic observations raises the possibility that upstream metabolic reprogramming contributes to these immune-modulating effects.

In parallel, natural compounds such as resveratrol inhibit HIF-1α stabilization in tumor cell lines and xenograft models, thereby reducing transcription of glycolytic enzymes and limiting hypoxia-driven tumor adaptation [116]. This is particularly relevant in HNSCC, where HIF-1α nuclear accumulation at invasion fronts drives *GLUT1/LDHA/PDK1* transcription, creating the Warburg phenotype that sustains both tumor growth and immune evasion. Resveratrol-mediated HIF-1α destabilization reduces *Ldha* expression and lactate output, providing a direct mechanistic link between metabolic intervention and immune microenvironment restoration in hypoxic tumors.

### 5.4. Lactate-Driven Immunosuppression

Lactate is increasingly recognized as an active immunosuppressive metabolite rather than a passive byproduct of glycolysis. Elevated tumor-derived lactate impairs cytotoxic T cell proliferation, reduces IFN-γ production, and stabilizes Tregs function, thereby reinforcing immune evasion. In melanoma mouse model, pharmacologic inhibition of lactate dehydrogenase A enhanced T cell function and improved responses to immune checkpoint blockade, underscoring the functional importance of lactate in tumor immunity [117]. Although direct lactate quantification in berry-treated HNSCC models remains limited, attenuation of glycolytic intermediates in BRB supplemented carcinogenesis models [20], together with suppression of Treg expansion [19], is consistent with a possible mechanistic link between metabolic modulation and attenuation of lactate-associated immunosuppressive features. Future studies integrating metabolic flux analysis, lactate quantification, and immune profiling will be essential to definitively establish this connection and to optimize metabolic targeting strategies using natural compounds.

## 6. Glucocorticoid Signaling at the Endocrine–Immune Interface

Glucocorticoids are central regulators of immune homeostasis, integrating endocrine signals with inflammatory and metabolic pathways. Through activation of the glucocorticoid receptor (GR), these steroid hormones exert potent anti-inflammatory and immunosuppressive effects by modulating transcriptional programs that control cytokine production, immune cell differentiation, and stress responses [118,119]. While systemic GCs are widely used clinically to suppress inflammation, their role within the TME is more complex and context-dependent, particularly in cancers such as HNSCC that are characterized by chronic inflammation and immune dysregulation. Emerging evidence indicates that local GC metabolism, rather than circulating hormone levels alone, critically shapes tumor-associated immune responses. This regulation is mediated in part by the bidirectional enzymes HSD11β1 and HSD11β2, which respectively activate and inactivate GCs at the tissue level. Dysregulation of this axis has been observed in multiple malignancies and may contribute to an immunosuppressive TME by altering local GC availability and GR signaling [120,121]. In HNSCC, recent studies demonstrate that GC metabolism is dynamically altered during tumorigenesis. Notably, reduced expression of HSD11β2, the enzyme responsible for inactivating GCs, has been observed in oral cancer tissues and experimental models, suggesting enhanced local GC signaling during tumor progression [21]. This shift may promote immune suppression by dampening pro-inflammatory signaling and impairing effective antitumor immune responses, thereby facilitating tumor growth.

Natural compounds including berry phytochemicals and their metabolic derivatives, have emerged as modulators of this endocrine–immune axis. Dietary BRB supplementation has been shown to restore HSD11β2 expression in oral carcinogenesis models, leading to reduced levels of active GCs within the TME [21]. This modulation of local GC metabolism was associated with suppression of tumor progression and alterations in inflammatory signaling, suggesting that phytochemicals may influence GC-driven immune regulation. Importantly, these effects extend beyond classical anti-inflammatory actions and implicate GC metabolism as a previously underappreciated target of dietary chemoprevention. Mechanistically, GC signaling intersects with multiple pathways discussed in preceding sections, including NF-κB mediated inflammation and metabolic regulators such as AMPK. However, rather than functioning as an isolated pathway, GC signaling acts as a higher-order regulatory node that integrates endocrine cues with immune and metabolic states. This integrative role positions GC metabolism as a critical determinant of whether the TME adopts an immunosuppressive or immunostimulatory phenotype. From a translational perspective, targeting local GC metabolism offers a novel strategy to modulate tumor-associated immune suppression without the systemic side effects associated with pharmacologic GC administration. Natural compounds that modulate GC signaling may therefore have potential as adjuncts to immunotherapeutic approaches by alleviating endocrine-driven immune suppression. In particular, modulation of the HSD11β1/HSD11β2 axis could enhance immune responsiveness and improve the efficacy of checkpoint blockade therapies.

Collectively, these findings highlight GC signaling as a critical but underexplored component of the tumor–immune microenvironment in HNSCC. By linking endocrine regulation with immune and metabolic reprogramming, this pathway provides a conceptual bridge between the mechanisms described in Section 4 and Section 5 and the therapeutic strategies discussed in subsequent sections. Further mechanistic and translational studies are needed to define how targeted modulation of GC metabolism can be leveraged for precision immunotherapy and chemoprevention.

## 7. Natural Compounds as Adjuncts to Immunotherapy

Through restoration of immune homeostasis via GC pathways, natural compounds may contribute to a microenvironment more permissive for combinatorial strategies with immune checkpoint inhibitors to improve clinical outcomes. Immune checkpoint blockade targeting PD-1/PD-L1 and CTLA-4 has transformed HNSCC therapy; however, only a subset of patients achieves durable responses, with many exhibiting primary or acquired resistance [122,123,124]. Resistance reflects multifactorial barriers, including persistent immunosuppressive signals, metabolic constraints, and Tregs dominance [84,125]. Berry-derived phytochemicals are being investigated as potential adjunctive agents that may influence several of these barriers through systems-level modulation. Rather than acting solely on checkpoint receptors, these compounds can complement PD-1/PD-L1 blockade by simultaneously reshaping metabolic and immune pathways. As mentioned, *Rubus coreanus* extract and ellagic acid of BRB have been reported to interfere with PD-1/PD-L1 interactions and reduces CTLA-4 expression on Tregs, enhancing CD8^+^ T cell cytotoxicity and reducing exhaustion [19,90]. Importantly, these effects extend beyond direct checkpoint modulation. Preclinical HNSCC models demonstrate that dietary BRB supplementation reduces tumor burden while modulating the tumor metabolic landscape, potentially alleviating metabolically mediated T cell suppression, and restoring effector T cell function [15,20,125]. These complementary actions provide a mechanistic rationale for synergy with checkpoint inhibitors, enhancing responsiveness and potentially reversing resistance. Translationally, incorporating standardized berry-derived formulations into neoadjuvant or adjuvant therapy could allow monitoring of immune and metabolic biomarkers including T cell activation, Treg frequency, DC maturation, lactate levels, and AMPK/mTOR signaling. Combination strategies with PD-1 inhibitors such as nivolumab or pembrolizumab in recurrent/metastatic HNSCC could potentially help in addressing resistance mechanisms by targeting complementary pathways. Beyond HNSCC, similar approaches may apply to other solid tumors with immunosuppressive TMEs, including NSCLC, melanoma, gastric cancer, and TNBC [126,127]. Overall, berry-derived phytochemicals function as context-modifying agents, not substitutes for checkpoint inhibitors, enhancing immunotherapy by alleviating immune and metabolic constraints. Future biomarker-driven clinical studies will be important for evaluating these hypotheses.

## 8. Translational Pharmacology of Berry-Derived Compounds: From Chemoprevention to Therapeutic Development

The growing evidence supporting the chemopreventive and immunomodulatory effects of berry-derived phytochemicals raises a central translational challenge on how these dietary agents can be advanced into clinically viable therapeutics. Addressing this requires a shift from observational efficacy to pharmacologic optimization, formulation engineering, and regulatory standardization. Unlike conventional single-target drugs, berry-derived compounds exhibit multi-component, multi-target activity, necessitating development strategies that integrate reductionist and systems-level approaches. Structurally, this complexity arises from structurally diverse phytochemical scaffolds (flavonoids, stilbenes, ellagitannins, and phenolic acids), where functional activity is governed by structural features such as hydroxylation patterns, glycosylation, degree of polymerization, and metabolite conversion, collectively defining their SAR in biological systems.

### 8.1. Whole Extracts Versus Isolated Molecules: Synergy, Reductionism, and Systems Pharmacology

A fundamental consideration in botanical drug development is whether to prioritize whole extracts or isolated bioactive compounds. Whole berry extracts contain complex mixtures of anthocyanins, ellagitannins, and phenolic acids that collectively modulate immune, inflammatory, and metabolic pathways. This phytochemical synergy may contribute to their observed chemopreventive activity in preclinical and clinical settings, including HNSCC models utilizing standardized BRB formulations [19,96].

In contrast, reductionist approaches focusing on individual compounds enable precise mechanistic interrogation and pharmacologic optimization. For example, PCA is a major anthocyanin metabolite, and it can exemplify this paradigm. PCA modulates DC activation, suppresses pro-inflammatory cytokine production, and regulates metabolic signaling pathways, reproducing several features of the biological activity observed with whole berry extracts [17,73]. Importantly, such metabolite-driven effects highlight that pharmacological activity in berry systems is often governed by bioactive transformation products rather than parent phytochemical structures, reinforcing a metabolite-centric structure–activity framework.

However, isolating single compounds may fail to capture emergent interactions among phytochemicals that contribute to efficacy in vivo. To address this, systems pharmacology approaches are increasingly employed to model multi-component interactions and network level effects. These frameworks enable the design of standardized yet biologically representative formulations, providing a framework for integrating phytochemical complexity and pharmacologic characterization. Collectively, whole extracts and isolated molecules should be viewed as complementary strategies, forming a continuum from biologically validated mixtures to optimized therapeutic agents.

### 8.2. Lead Optimization Strategies

Based on these foundational considerations, translation into therapeutics requires optimization of stability, potency, and delivery. Natural compounds frequently exhibit poor bioavailability, rapid metabolism, and limited pharmacologic exposure, necessitating targeted refinement.

Structural analog development and stabilization represent key approaches to enhance drug-like properties. Anthocyanins, while biologically active, are chemically unstable under physiological conditions. Stabilization strategies, including molecular complexation, have demonstrated improved functional longevity. For example, complex formation with yeast mannoproteins significantly enhanced anthocyanin thermal stability, extending half-life under stress conditions [128]. At the molecular level, stabilization strategies aim to preserve or modify key functional motifs such as hydroxylation patterns and conjugated ring systems that are essential for interaction with signaling proteins including EGFR, PI3K, and NF-κB pathway components.

In parallel, nanoparticle-based delivery systems have emerged as useful approaches to overcome pharmacokinetic limitations. Lipid-based nanoparticles enhance intracellular delivery and preserve functional activity, as demonstrated in HNSCC models using miRNA delivery platforms [129]. Similarly, co-encapsulation strategies combining phytochemicals with chemotherapeutic agents, such as resveratrol with docetaxel, improve bioavailability and produce synergistic antitumor effects [130].

Another translationally relevant approach involves standardized botanical formulations, which retain phytochemical complexity while ensuring reproducibility. Freeze-dried BRB preparations have demonstrated consistent chemopreventive efficacy in preclinical models [19] and have been evaluated in early-phase clinical studies, where standardized formulations modulated tumor-associated biomarkers in patients with oral cancer. These findings support a hybrid translational model in which chemically defined optimization and biologically complex formulations are not mutually exclusive but represent complementary stages of phytochemical drug development [96].

### 8.3. Translational Barriers

Despite these advances, several challenges must be addressed to enable clinical deployment. A major limitation is oral mucosal bioavailability, particularly relevant in HNSCC. The stratified squamous epithelium and continuous salivary clearance limit compound retention and absorption [131]. Mucoadhesive delivery systems, such as chitosan-based nanoparticles, enhance mucosal residence time and penetration, achieving significantly improved tissue uptake and biological activity [132].

Pharmacokinetic constraints further limit therapeutic efficacy. Anthocyanins and ellagic acid exhibit rapid absorption and clearance, with peak plasma levels occurring within 1–2 h and systemic recovery typically less than 1% of the administered dose [133]. These findings highlight the need for improved delivery and retention strategies. At a molecular pharmacology standpoint, these limitations are directly linked to structural instability, rapid phase II conjugation, and microbiota-dependent degradation of polyphenolic scaffolds, which collectively reduce systemic exposure of parent compounds.

An additional layer of complexity arises from microbiome-dependent metabolism, which governs the conversion of dietary phytochemicals into bioactive metabolites such as PCA. Variability in gut microbiota composition contributes to interindividual differences in therapeutic response.

Finally, regulatory and dosing challenges remain significant. Berry-derived compounds occupy a regulatory space between dietary supplements and botanical drugs, each with distinct requirements. Moreover, defining pharmacodynamically relevant dosing and validated biomarkers of response is essential for clinical translation.

Together, these advances define a translational pipeline in which berry-derived phytochemicals evolve from complex dietary agents into optimized, standardized, and potential therapeutic platforms warranting further clinical investigation. Integrating systems pharmacology, delivery engineering, and regulatory strategy is critical to fully realize their potential in cancer prevention and treatment. The translational relevance of these compounds, including their synergistic interactions with conventional therapies and immunotherapy, is further summarized in Table 1 and supports their application in precision natural drug discovery.

## 9. Future Perspectives in Precision Natural Drug Discovery

**Figure 2:**
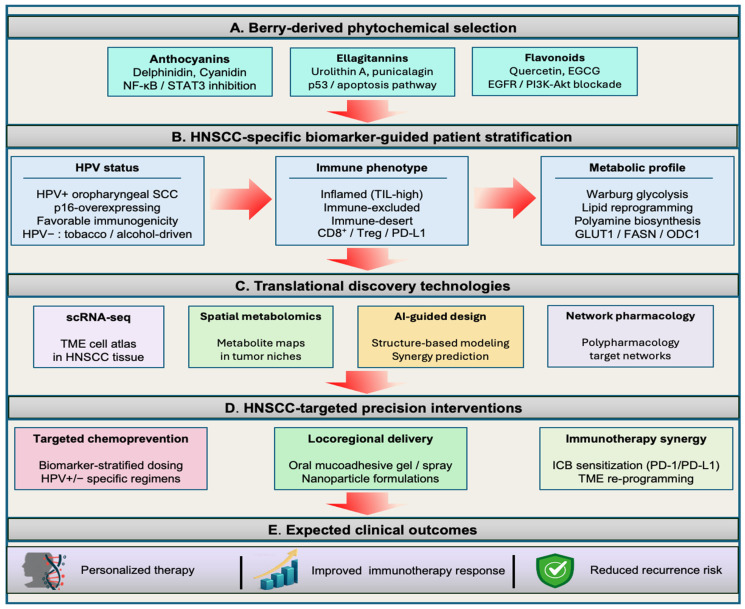
Conceptual framework for precision-oriented development of berry-derived compounds in HNSCC. Biomarker-guided precision chemoprevention framework for berry-derived phytochemicals in HNSCC, integrating HPV status, immune phenotype, and metabolic profiling to direct compound selection, delivery strategy, and immunotherapy co-treatment.

Optimized berry phytochemicals may support future patient-tailored strategies that exploit immune, metabolic, and molecular profiling to guide chemoprevention and therapy in HNSCC. Emerging tools including multi-omics, artificial intelligence (AI)-driven discovery, and innovative local delivery are converging to support emerging precision-oriented approaches to natural product discovery paradigm with translational impact. As shown in Figure 2, our precision natural drug discovery framework integrates patient stratification, multi-omics technologies, and phytochemical-based compound selection to guide individualized interventions in HNSCC.

### 9.1. Precision Chemoprevention

Precision chemoprevention leverages molecular, immune, and metabolic stratification to guide intervention selection. Berry-derived phytochemicals, abundant in anthocyanins, ellagitannins, ellagic acid, and stilbenes, exhibit anti-inflammatory, antioxidant, and antiproliferative activity as discussed above [134]. Stratification strategies include the following.

#### 9.1.1. HPV Status

HPV-positive tumors exhibit distinct oncogenic programs via viral E6/E7-mediated p53 and RB suppression, altered immune infiltrates, and metabolic dependencies. Targeted phytochemicals, such as ellagic acid, have been reported to suppress HPV-driven oncogenic signaling and oxidative stress pathways [15,135].

#### 9.1.2. Immune Phenotype

Tumors can be classified as immune-inflamed, immune-excluded, or immune-desert, with implications for therapeutic responsiveness. Berry-derived anthocyanins enhance CD8^+^ T cell infiltration and reduce Treg accumulation in preclinical models [136], supporting the concept of immune guided chemoprevention strategies.

#### 9.1.3. Metabolic Signatures

HNSCC exhibits metabolic reprogramming, including aerobic glycolysis, altered lipid metabolism, and mitochondrial dysfunction. Compounds such as berberine modulate AMPK, suppress glycolysis, and restore mitochondrial function, suggesting a potential metabolic-oriented precision approach [137]. Integration of metabolomic profiling from saliva or oral mucosa may guide patient-specific phytochemical selection.

These stratification layers collectively form the foundation for precision chemoprevention and are summarized in Figure 2.

### 9.2. Localized Delivery Innovation

Poor systemic bioavailability of phytochemicals limits clinical translation. Localized delivery strategies enhance oral and oropharyngeal bioavailability while minimizing systemic exposure.

#### 9.2.1. Mucoadhesive Gels

Bioadhesive polymers prolong compound retention at the mucosa. BRB gels reduce dysplasia grade, modulate NF-κB signaling, and suppress proliferation in clinical trials [138].

#### 9.2.2. Topical Oral Formulations

Mouth rinses, sprays, and lozenges enable repeated site-specific dosing. Anthocyanin and ellagic acid formulations achieve dose-dependent mucosal penetration [139].

#### 9.2.3. Controlled Release Systems

Nanoparticles, liposomes, and biodegradable polymers enhance stability, uptake, and sustained release. Cyanidin-3-glucoside chitosan nanoparticles demonstrate improved cellular internalization and antiproliferative activity in oral squamous carcinoma models [140]. Targeted delivery strategies, for example, folate or EGFR-conjugated carriers, can further refine lesion-specific selectivity. As illustrated in Figure 2, localized delivery strategies may facilitate translational bridge between compound selection and effective precision intervention in HNSCC.

### 9.3. Immunometabolic Target Mapping

Advances in multi-omics technologies are enabling high-resolution mapping of tumor ecosystems and identification of actionable targets. Figure 2 further highlights how multi-omics technologies support immunometabolic target mapping and identification of patient-specific vulnerabilities.

#### 9.3.1. Single-Cell Transcriptomics

Single-cell RNA sequencing reveals heterogeneity across malignant, immune, and stromal compartments in HNSCC [23]. Importantly, integrating phytochemical perturbation datasets with single-cell resolution profiling enables identification of cell-type specific responses, thereby distinguishing direct tumor effects from immune-mediated mechanisms. This approach can also uncover resistance pathways and adaptive cellular states that emerge following exposure to berry-derived compounds.

#### 9.3.2. Spatial Metabolomics

Imaging-based metabolomics enables spatial mapping of metabolic activity within tumor tissues, preserving microenvironmental context. This is particularly relevant for phytochemicals with low systemic bioavailability, where local metabolite distribution rather than parent compound levels drives biological activity. Spatial metabolomics allows tracking the intratumoral distribution of anthocyanin metabolites, including quinic acid, methylsuccinic acid, chlorogenic acid, oxadipic acid, and malic acid derivatives, and to correlate their spatial distribution with local metabolic reprogramming events [141]. Such analyses provide direct evidence linking metabolite presence to functional modulation within specific tumor niches.

#### 9.3.3. Network Pharmacology

Phytochemicals exhibit polypharmacology across interconnected signaling networks. Systems-level analyses have identified convergence on key regulatory hubs such as TP53, EGFR, and PI3K/AKT pathways [142]. Rather than acting as single-target inhibitors, berry-derived compounds and their metabolites function as network modulators, reinforcing pathway level regulation across immune, metabolic, and stress response axes.

These approaches collectively advance mechanism-driven, systems-level phytochemical targeting and are integrated within the precision framework shown in Figure 2.

### 9.4. AI-Driven Phytochemical Discovery

Artificial intelligence is accelerating natural product drug discovery by enabling scalable analysis of chemical and biological complexity. AI-driven phytochemical discovery further expands this framework by improving target prediction, synergy modeling, and biomarker-guided therapeutic design, as summarized in Figure 2.

#### 9.4.1. Structure-Based Modeling

Advancement in machine learning algorithms and structural bioinformatic tools are increasingly applied to predict the binding interactions between berry phytochemicals and oncogenic protein targets implicated in HNSCC pathogenesis. Deep learning-based binding affinity models have been applied to screen anthocyanin libraries against MAPK1, MMP-9, and PIK3CA structural models, identifying cyanidin and delphinidin derivatives as high affinity predicted binders at oncogenic target sites [143]. Given the limited in vivo stability of parent anthocyanins, emerging models are now incorporating metabolite libraries (e.g., PCA, urolithins), improving biological relevance of computational predictions. AlphaFold2 guided protein structure prediction can enable modeling of previously intractable HNSCC targets including mutant p53 conformers and intrinsically disordered signaling proteins expanding the computational target space accessible to natural compound virtual screening campaigns [144]. These computational strategies substantially accelerate hit identification and enable mechanistic hypothesis generation prior to resource-intensive experimental validation.

#### 9.4.2. Synergy Prediction

Phytochemicals frequently demonstrate synergistic activity when combined with complementary natural agents or conventional cytotoxic therapies, offering potential to enhance therapeutic efficacy while mitigating toxicity. AI-based combinatorial prediction models trained on large scale drug interaction datasets including NCI ALMANAC and DrugComb can systematically evaluate multi-dimensional combinatorial spaces to identify berry phytochemical combinations with high predicted synergistic indices [145]. This is particularly important for multi-component berry extracts, where emergent activity arises from combined metabolite interactions rather than isolated compounds. For example, a polyphenolic compound, luteolin alone and in combination with chemotherapeutic agents have demonstrated synergistic antiproliferative activity in cancer cell line models, indicating AI augmented screening could systematically expand the discovery of such combinations across the broader berry metabolome [146].

#### 9.4.3. Biomarker-Guided Design

AI-driven integrative analysis of multiomic datasets encompassing genomic, transcriptomic, epigenomic, proteomic, and metabolomic data can enable the identification and validation of molecular biomarkers predictive of response to specific phytochemical interventions. Candidate predictive biomarkers for natural compound responsiveness may include metabolizing enzyme expression profiles, gut-microbiome-dependent urolithin-producing capacity, FASN overexpression as a determinant of ellagic acid sensitivity, and NRF2 pathway activation status as a modulator of anthocyanin antioxidant response. This framework supports the development of biomarker stratified phytochemical therapy, wherein patients are assigned to targeted berry compound regimens based on their individual molecular and microbiome profiles, analogous to companion diagnostic approaches established in conventional precision oncology. Prospective validation of AI-identified biomarker signatures in clinical cohorts will be essential to realize the full translational potential of this paradigm.

Taken together, precision natural drug discovery integrates molecular stratification, innovative delivery, immunometabolic mapping, and AI-guided compound optimization into a cohesive framework for HNSCC prevention and therapy (Figure 2). A key unifying principle across these approaches is the recognition that phytochemical efficacy is governed by dynamic metabolic transformation, tissue distribution, and systems-level interactions rather than single compound activity. Translational success requires standardized extract characterization, validated biomarker pipelines, and regulatory alignment. Coordinated clinical evaluation, building upon early-phase BRB trials, can establish phytochemical-based precision oncology as a feasible, evidence-driven component of HNSCC management.

## 10. Conclusions

HNSCC remains a heterogeneous and immunosuppressive malignancy with limited durable responses to current therapies. This underscores the need for complementary strategies that address the interconnected immune, metabolic, and signaling networks underlying disease progression. Berry-derived phytochemicals represent a class of multi-target compounds that have been reported to modulate pathways implicated in HNSCC, including inflammatory signaling, immunoregulation, and metabolic reprogramming. Preclinical and early clinical studies suggest biological activity relevant to inflammation, immune regulation, and tumor-associated signaling; however, their clinical translation is constrained by challenges related to bioavailability, pharmacokinetics, and formulation variability.

Advances in translational pharmacology including structural optimization, nanoparticle-based delivery, and standardized botanical formulations may improve the stability and delivery of these compounds. These developments have increased interest in mechanism-driven therapeutic investigation beyond observational chemoprevention. Emerging precision frameworks further expand this potential. Integration of molecular stratification (e.g., HPV status, immune phenotype, and metabolic signatures) with multi-omics profiling, spatial analysis, and AI-guided discovery could facilitate more targeted and context-specific application of natural compounds. In parallel, localized delivery approaches in the oral cavity may help to enhance local tissue exposure while minimizing systemic limitations. Although early-phase studies, particularly with berry-derived formulations, suggest feasibility and biological activity, rigorous preclinical validation and well-designed clinical trials are required to establish efficacy, optimize dosing, and define appropriate clinical settings. Overall, natural compounds may have potential as adjunctive components within integrative, precision-oriented strategies for HNSCC, with potential to complement existing prevention and treatment approaches when guided by mechanistic and patient-specific insights. Future studies integrating phytochemical structural characterization with mechanistic and translational oncology approaches may facilitate the development and evaluation of berry-derived compounds in HNSCC prevention and therapeutic research.

## Data Availability

No new data were created or analyzed in this study. Data sharing is not applicable to this article.
